# Lysosomal protease-mediated APP degradation is pH-dependent, mutation-sensitive, and facilitates tau proteolysis

**DOI:** 10.1186/s44477-025-00017-6

**Published:** 2026-01-19

**Authors:** Caroline Ackley, Zoe Liau, Shruti Arya, Tara Antee, Emily Cheang, Giselle M. Knudsen, Courtney Lane-Donovan, Paul J. Sampognaro, Aimee W. Kao

**Affiliations:** 1 Edward and Pearl Fein Memory and Aging Center, Department of Neurology, University of California San Francisco, San Francisco, 100190 California USA; 2 Neuromuscular Division, Department of Neurology, University of California San Francisco, San Francisco, 100190 California USA; 3 Alaunus Biosciences, Inc., South San Francisco, 94080 California USA

**Keywords:** Amyloid-precursor protein, APP, Cathepsin, Protease, Amyloid-beta, A$\beta$, Alzheimer’s disease, Lysosome, Autophagy, Tau, Neurodegeneration

## Abstract

**Background:**

The accumulation and aggregation of amyloid beta (A$\beta$)—a peptide fragment derived from the proteolytic processing of amyloid precursor protein (APP)—is a central pathological feature of Alzheimer’s disease (AD) and a current target for disease-modifying therapies. Mutations in APP can also drive early-onset AD. While the roles of $\alpha$-, $\beta$-, and $\gamma$-secretases and their respective cleavage sites in APP processing are well characterized, much less is understood about the routine degradation of APP within sub-cellular compartments like the lysosome.

**Methods:**

We applied Multiplexed Substrate Profiling by Mass Spectrometry (MSP-MS) to map cleavage sites within APP that may be targeted by lysosomal proteases, also known as cathepsins. We then employed cell-based and in vitro assays to examine the degradation of both wild-type and mutant APP by these enzymes.

**Results:**

Our findings confirm that APP is enriched in the endo-lysosomal compartment, where it is processed by many cathepsins. Our experiments reveal that cleavages at several mapped APP sites are sensitive to both changes in pH and the presence of pathogenic variants E693G and E693Q. Additionally, we discovered that the large soluble domain of APP (sAPP) enhances tau cleavage by a specific cathepsin, CTSG, in vitro.

**Conclusions:**

Collectively, these results underscore the importance of lysosomal processing of APP, identify a link between APP and tau, and suggest new avenues for exploring AD pathogenesis. They also highlight potential therapeutic targets related to the lysosomal function of APP and its impact on neurodegenerative diseases.

## Background

### Proteolytic processing of APP in Alzheimer’s disease

Proteolytic processing of the amyloid-beta precursor protein (APP) is a central event in Alzheimer’s disease pathogenesis. Although extensive studies have characterized A$\beta$ generation via secretases and caspases [1–6], the specific mechanisms by which APP processing contributes to neurodegeneration are not fully understood. To date, research has predominantly focused on a relatively narrow 37–49 amino acid segment of APP—the amyloid-beta (A$\beta$) peptide—even though full-length APP contains up to 770 amino acids (depending on the isoform) and full-length APP has been identified within amyloid plaques on autopsy [7]. Moreover, while cell surface APP is known to play crucial roles in neurodevelopment and synaptic plasticity [8], the functions and processing of the non-amyloid portions of APP within neurons remain largely unexplored. While APP is known to accumulate and undergo cleavage into A$\beta$ and other fragments within the endolysosomal system [9–17], a comprehensive characterization of its processing by lysosomal proteases is still lacking. Therefore, we set out to map the cleavage sites of these enzymes as part of efforts to investigate novel mechanisms of A$\beta$ release, shed light on the broader cellular processing of APP, and potentially open new avenues for understanding and treating AD and related conditions.

### Lysosomal proteases and neurodegeneration

A common hallmark of many neurodegenerative diseases, including AD, is the accumulation of misfolded proteins. Lysosomes are key degradative organelles in cells, distinguished by their highly acidic luminal pH. Regarded as the “recycling centers” of cells, lysosomes are critical for autophagy, protein homeostasis, and the clearance of protein aggregates resulting from such misfolded proteins. The proteolytic capacity of lysosomes is mediated by a diverse family of proteases known as cathepsins, which includes serine, aspartyl, and cysteine proteases, all of which exhibit distinct substrate preferences that are dependent upon local pH [18]. Disruptions in lysosomal function—whether through alterations in pH [19–22] or genetic variants affecting lysosomal components [23, 24]—have been strongly implicated in neurodegeneration. Notably, an overabundance of APP fragments has also been shown to impede normal lysosomal activity [25–28]. Despite these associations, our understanding of how lysosomal proteases process and degrade proteins associated with neurodegenerative diseases remains incomplete. This gap is particularly critical given that impaired lysosomal proteolysis could exacerbate the accumulation of pathogenic proteins and thereby accelerate disease progression.

### A novel approach to mapping APP cleavage

To address this critical gap, we employed Multiplexed Substrate Profiling by Mass Spectrometry (MSP-MS) on a custom-designed library of APP-derived peptides. By incubating these peptides with a panel of 13 lysosomal proteases—including 12 cathepsins and asparagine endopeptidase (AEP)—under varying pH conditions, our approach allowed us to delineate cleavage “hot spots” within APP as well as regions with low proteolytic susceptibility (e.g., the flexible acidic linker between the E1 and E2 domains). This comprehensive mapping not only revealed enzyme-specific cleavage patterns (with some sites being pH-dependent) but also provided predictive insights into how efficiently different proteases degrade soluble APP (sAPP). In particular, our data showed that proteolytic efficiency decreases with increasing pH, underscoring the importance of the lysosomal microenvironment in APP processing.

### Linking APP processing to broader neurodegenerative mechanisms

Building upon our APP cleavage map, we compared these results with our previously published datasets on tau, TDP-43, and $\alpha$-synuclein. Integrating these datasets enabled us to predict how pathogenic APP variants might alter lysosomal processing. Previously, we found that several pathogenic variants in tau, TDP-43, and alpha-synuclein reduce their rate of cleavage by cathepsin proteases [29]. Contrary to our initial hypothesis that deleterious variants would impede APP clearance, we observed that the Dutch (E693Q) and Arctic (E693G) variants paradoxically enhanced proteolysis by lysosomal enzymes relative to the reference protein. This accelerated clearance was further corroborated in differentiated SH-SY5Y cells engineered to express mutant or wild-type APP. Moreover, we found that sAPP selectively promoted tau degradation in vitro, an unexpected finding that suggests a previously unrecognized role for APP in modulating lysosomal proteolysis, as well as the clearance of tau. Taken together, these findings build upon specific aspects of the Amyloid Cascade Hypothesis (ACH) and point towards novel therapeutic strategies targeting lysosomal processing pathways that may hold promise for modulating both APP and tau pathology in AD.

## Methods

### Recombinant proteases

Cathepsin A (CTSA) (R&D #1049-SE), Cathepsin B (CTSB) (Millipore #219,364), Cathepsin C (CTSC) (R&D #1071-CY), Cathepsin D (CTSD) (R&D #10140AS) for MSP-MS; (Sigma Aldrich #C8696) for all other assays, Cathepsin E (CTSE) (R&D #1294-AS), Cathepsin F (CTSF) (Abcam #ab240858), Cathepsin G (CTSG) (Millipore #219,873), Cathepsin H (CTSH) (R&D #7516-CY-010), Cathepsin K (CTSK) (Millipore #219,461), Cathepsin L (CTSL) (Millipore #219,402), Cathepsin O (CTSO) (Abcam #ab267932), Cathepsin S (CTSS) (R&D #1183-CY), Cathepsin V (CTSV) (R&D #1080-CY), Cathepsin X (CTSX) (R&D #934-CY), and asparagine endopeptidase (AEP) (R&D #2199-CY).

### Antibodies

The following primary antibodies (commercial identifier, dilution) were used: 1) monoclonal rabbit anti-APP (Abcam #ab32126, 1:500), 2) monoclonal mouse anti-APP 22C11 (Invitrogen #14–9749−80, 1:1000, Fig. 1C), 3) monoclonal mouse anti-ATP5A1 (Invitrogen #459240, 1:500), 4) monoclonal rabbit anti-CTSB (Abcam #ab214428, 1:1000), 5) monoclonal rabbit anti-CTSD (Abcam #ab75852, 1:1000), 6) monoclonal mouse anti-EEA1 (BD #610457, 1:100), 7) monoclonal mouse anti-GAPDH (Abcam #ab8245, 1:1000), 8) polyclonal rabbit anti-GAPDH (Abcam #ab9485), 9) monoclonal mouse anti-HA (Invitrogen #26183, 1:500), 10) monoclonal mouse anti-Lamp1 (BioLegend #328611, 1:500, Fig. 1A), 11) monoclonal rabbit anti-Lamp1 (Cell Signaling #9091S, 1:1000, Fig. 1C), 13) monoclonal mouse anti-Lamp2 (DSHB #H4B4, 1:100), 14) monoclonal rabbit anti-Calreticulin (Cell Signaling, #12238, 1:1000), 15) monoclonal mouse anti-ATP5A1 (Invitrogen, #459240, 1:1000), 16) monoclonal mouse anti-tau (Tau 46; Santa Cruz Biotechnology, #sc-32274, 1:1000), 17) monoclonal mouse anti-tau (Tau 5; Millipore, #MAB361, 1:1000), and 18) monoclonal mouse anti-tau (HT7; Thermo Scientific, #MN1000, 1:1000).

The following secondary antibodies were used for confocal microscopy: 1) polyclonal goat anti-rabbit AF488 (Thermo Scientific #A11008, 1:500, SH-SY5Y immunohistochemistry), 2) polyclonal goat anti-mouse AF647 (Life Tech #A21236, 1:500, SH-SY5Y immunohistochemistry), 3) polyclonal donkey anti-goat AF647 (Life Tech #A21447, 1:500, mouse cortex immunohistochemistry), 4) polyclonal donkey anti-mouse AF555 (Life Tech #A31570, 1:500, mouse cortex immunohistochemistry), and 5) polyclonal donkey anti-rabbit AF488 (Life Tech #A21206, 1:500, mouse cortex immunohistochemistry).

### Generation of stable SH-SY5Y cell lines (APP WT, E693G, E693Q)

To generate stable APP-expressing SH-SY5Y lines, we first designed and synthesized a doxycycline-inducible, FLAG-tagged, full-length wild-type APP lentiviral construct using a puromycin-resistant plasmid backbone (pTet-O-Ngn2-Puro, Addgene #52047; Epoch Life Sciences). Starting from this wild-type construct, additional plasmids bearing single pathogenic point mutations (E693G and E693Q) were created. Lentivirus was produced using psPAX2 (Addgene #12260) and pCMV-VSV-G (Addgene #8454) packaging and envelope plasmids, respectively. SH-SY5Y cells already containing the pLenti CMV rtTA3 Blasticidin construct (Addgene #26429) were infected and selected with 1 mg/mL puromycin. This resulted in three stable cell lines expressing either wild-type or mutant APP.

### Animals

All mouse studies were conducted in accordance with the NIH guidelines and approved by the Institutional Animal Care and Use Committee (IACUC) of the University of California, San Francisco. To generate neuron-specific LysoTag mice, B6.129S4-Gt(ROSA)26Sortm1(CAG-TMEM192)Dmsa/J [30] mice were crossed with B6.Cg-Tg(Syn1-cre)671Jxm/J mice. These animals, maintained as heterozygotes, were used for all experiments. Mice were anesthetized with isoflurane for tissue collection. Perfusion with PBS followed by 4% paraformaldehyde was performed for immunohistochemistry, while fresh tissues for immunoprecipitation were collected after isoflurane sacrifice.

### Immunohistochemistry (IHC)

Mouse cortex and cerebellum tissue was cryopreserved and sectioned into 30 $\mu$m free-floating cortical slices using a cryostat. Sections underwent antigen retrieval in pH 8 Tris-EDTA at 80 $^{\circ }$C for 30 minutes prior to blocking and antibody incubation.

Immunostaining of SH-SY5Y cells was performed in 8-well chamber slides (Ibidi #80841). Cells were examined either one day after plating (undifferentiated, Figure S1) or following a 10-day differentiation protocol with retinoic acid and BDNF [31]. They were then fixed in 4% paraformaldehyde for 15 minutes, washed with 0.1% Triton X-100 in PBS, and subsequently incubated with primary and secondary antibodies. Following antibody staining, all samples were sealed with Fluoroshield Mounting Medium with DAPI (Abcam #ab104139) and a coverslip.

#### Microscopy

Following IHC, cells were imaged using a Nikon CSU-W1 spinning disk confocal microscope in the Nikon Imaging Center (NIC) at UCSF. Images were captured using a Plan Apo VC 100x/1.4 numerical aperture oil immersion lens and Andor Zyla sCMOS camera. DAPI, AF488, and AF647 fluorophores were stimulated using 405, 488, and 640 nm wavelength excitation, respectively.

#### Colocalization analysis

Colocalization analysis of APP with organelle markers was performed using ImageJ/FIJI software using a previously described method [32]. Briefly, a threshold was set for both APP and organelle marker channels to isolate the brightest 2% of pixels, and each remaining area was measured. The overlapping area was then calculated and divided by the thresholded APP area to determine the percentage APP colocalization with each marker (Figure S1).

### Immunoprecipitation (IP)

LysoTag mice were sacrificed and fresh brain tissue collected for immediate IP as described previously [30]. Briefly, fresh brain tissue (cortex or cerebellum) was immediately homogenized in cold PBS using a glass dounce homogenizer (20 strokes). A portion of the homogenate was saved as a whole-cell control, while the remainder was mixed with 150 $\mu$L of prewashed anti-HA magnetic beads (BioLegend #901501) and rotated at 4 $^{\circ }$C for 20 minutes. Lysosomal fractions were separated using a magnetic rack, then resuspended in 1% NP-40 in PBS. After 10 minutes of rotation at 4 $^{\circ }$C to remove beads, protein concentration was determined by BCA assay, and samples were stored at −80 $^{\circ }$C for subsequent Western blot analysis. Based on BCA analysis, 1–5% of immunoprecipitated product was loaded onto each gel, relative to $\sim$0.02% of flow through (“Flow Thr”) and 0.6% of whole cell lysate.

### Lysosome isolations

SH-SY5Y cells were differentiated as previously described [33, 34]. Cells were harvested 14 days post-differentiation and their lysosomes were isolated using a gradient-ultracentrifuge based technique (Lysosome Enrichment Kit for Tissues and Cultured Cells from Thermofisher, #89,839). Successful isolation and enrichment of lysosomes were confirmed by Western Blot using a Lamp1 antibody.

### Silver stains

Proteolytic assays were performed by incubating 1 $\mu$g of recombinant human full-length APP/Protease Nexin II (R&D Systems #3466-PI) or a protease-specific control substrate with or without 1 $\mu$M of individual proteases. CTSA, CTSC, CTSH, and CTSX were pre-activated according to the manufacturer’s instructions. Reactions were carried out for 1 hour at 37 $^{\circ }$C in a total volume of 19.5 $\mu$L using buffer conditions that included: 100 mM sodium citrate (pH 3.4), 50 mM sodium acetate (pH 4.5 or 5.5), or 100 mM PBS (pH 7.4), supplemented with 1 mM EDTA and 2 mM DTT. Protease activity was halted by adding 7.5 $\mu$L of NuPAGE 4X LDS (Fisher Scientific #NP0007) and 3 $\mu$L of 10X reducing agent (50 $\mu$M) (Fisher #NP0009), followed by denaturation at 80 $^{\circ }$C for 10 minutes. Samples were resolved using a NOVEX NuPAGE 4–12% Bis-Tris gel (Fisher #NP0321PK2) in MES buffer (Fisher #NP0002). After electrophoresis, the gel was fixed (40% ethanol, 10% acetic acid) and silver-stained according to the manufacturer’s instructions (Thermo Fisher #LC6070).

### Multiplexed substrate profiling by mass spectrometry (MSP-MS)

MSP-MS was performed as previously described by [35] as well as our previous work [29]. First, a library of 18 amino acid-long peptides was created to cover the entire APP-770 sequence (Uniprot accession number P05067-1) with roughly 5 amino acid overlaps between fragments. To prevent aggregation resulting from disulfide bridges between peptides, all Cys residues were converted to Ala. Additional residues were added to both termini of each peptide to allow for liquid chromatography tandem mass spectrometric (LC-MS/MS) analysis. A complete table of peptides used in this library is provided in (Table S1).

Lysosomal proteases were then incubated at 37 $^{\circ }$C with the entire peptide library at 500 nM per peptide. Protease concentration ranged between 1–70 nM, with the exception of CTSF at 1 $\mu$M. Reactions were monitored at 30, 60, and 240 minutes in an endpoint screening format. Aliquots were immediately desalted with C18 zip tips (Millipore-Sigma) and then freeze-dried. Samples were re-suspended in 0.1% formic acid in HPLC-grade water followed by LC-MS/MS analysis. For CTSA, which was pre-activated with CTSL, a matched CTSL-only negative control was compared for cleavage identification as described previously (Table S2).

### Mass spectrometry

Peptide sequencing by LC–MS/MS was performed on a QExactive Plus mass spectrometer (Thermo) equipped with a nanoACQUITY (Waters) ultraperformance LC (UPLC) system and an EASY-Spray ion source (Thermo). Reversed-phase chromatography was carried out with an EASY-Spray PepMap C18 column (Thermo, ES800; 3 $\mu$m bead size, 75 $\mu$m by 150 mm). Chromatography was performed at a 600-nL/min flow rate during sample loading for 14 min and then at a 400-nL/min flow rate for peptide separation over 90 min with a linear gradient of 2 to 35% (vol/vol) acetonitrile in 0.1% formic acid. Peptide fragmentation was performed by higher-energy collisional dissociation (HCD) on the six most intense precursor ions with a minimum of 2,000 counts, with an isolation width of 2.0 m/z and a minimum normalized collision energy of 25. Data were analyzed using Protein Prospector software, v.6.2.1 (http://prospector.ucsf.edu/prospector/mshome.htm, UCSF) using published methods [35]. The peptide cleavage data were then output as 8-mer sequences that spanned the P4–P4’ sites for each verified cleavage site (Supplemental Data).

### In silico analysis of MSP-MS data

To identify specific cleavage sites following MSP-MS, enzyme-treated sample results were subtracted from a no-enzyme control incubation. Unique cleavage sites for each enzyme (i.e. all identified P1 sites for any pH condition or endpoint) were mapped for further analysis in R and through the generation of IceLogos. A Pearson correlation was performed to measure the degree of cleavage similarity between enzymes. The strength of each correlation with corresponding significance values were then plotted as a heatmap. A Euclidean distance matrix calculation followed by unsupervised hierarchical clustering was performed to compare cleavage patterns between APP and other proteins previously analyzed using MSP-MS [29].

### Protease activity assays

Protease activity assays were performed in triplicate either in black, flat-bottom 384-well plates (Corning #3544) using custom designed fluorescent APP peptide substrates and FITC tau-441 (rPeptide #T-1113-1) or in Eppendorf Protein LoBind Tubes (Fisher Scientific #E925000090) using Recombinant Human APP (R&D Systems #3466PI010) and Recombinant Human 2N4R tau-441 (VWR #103790-836). Assays were conducted at 37 $^{\circ }$C for six hours (fluorogenic assays) or 30 minutes (LoBind tube assays). The following buffers, each supplemented with 2 mM DTT and 1 mM EDTA, were used: 100 mM sodium citrate (pH 3.4), 50 mM sodium acetate (pH 4.5 or 5.5), or 50 mM phosphate-buffered saline (pH 7.4). In fluorogenic assays, final concentrations were as follows: 20 nM cathepsins, 20 $\mu$M APP peptide substrates, 400 nM FITC-labeled tau-441, 5 $\mu$M recombinant human tau-441, and 20–50 nM of APP or BSA. Concentrations used in in vitro assays followed by Western blot were as follows: 10 nM CTSG, 10 nM CTSL, 20 nM CTSD, 50 nM sAPP, 50 nM BSA, and 500 nM 2N4R tau-441.

#### Fluorogenic APP peptide substrates

Each fluorescent APP or tau peptide substrate consisted of an 8-residue sequence flanked by a 7-methoxycoumarin-4-acetic acid (MCA) fluorophore at the N-terminus and a dinitrophenol (DNP)-modified lysine quencher at the C-terminus (Table S3). To enhance solubility, two arginine residues were included within each substrate. Before cleavage, the MCA signal is quenched by DNP. Proteolytic cleavage separates the fluorophore and quencher, resulting in detectable fluorescence. MCA fluorescence was measured using a BMG Labtech VANTAstar plate reader (excitation: 328 nm, emission: 393 nm).

#### FITC tau-441

FITC tau-441 is a commercially available, full-length, recombinant, mature human tau isoform that is covalently labeled with Fluorescein Isothiocyanate (rPeptide #T-1113). Similar to the fluorogenic APP peptide substrates, it does not fluoresce when intact but becomes fluorescent when cleaved by protease activity. FITC fluorescence was monitored with a BMG Labtech VANTAstar plate reader using excitation and emission wavelengths of 485 nm and 530 nm respectively.

### Western blots

For Western blot analysis, samples were denatured as described above and separated on pre-cast 4–12% NOVEX NuPAGE Bis-Tris gels using MOPS running buffer (Fisher Scientific #NP0001) and Invitrogen Novex Sharp Pre-Stained Protein Standards (Fisher Scientific #LC5800). Membranes were blocked at room temperature with Odyssey Blocking Buffer (LI-COR #927-40,100) and incubated at 4 $^{\circ }$C overnight with primary antibodies and 1 hour at room temperature with fluorescent secondary antibodies (LI-COR, 1:5000). Immunoreactive bands were visualized using a LI-COR Odyssey CLx image scanner and quantified with Image Studio version 5.5.

### Statistical analysis

Details of the statistical test used for each experiment is described in figure legends along with sample size and significance. All data is represented as mean ± standard error. Statistical analysis for plate reader assays was performed using GraphPad Prism 9 (GraphPad Software, La Jolla, California USA) while all other analysis was performed in R. Western blot quantifications and ELISA results were analyzed using two-way ANOVA. For all tests, a *p*-value < 0.05 was considered significant.

#### Heirarchical clustering analysis

Heirarchical clustering was performed to compare the relative contributions of each enzyme in cleaving APP (determined through MSP-MS) with our previously published analysis of tau, $\alpha$-syn, and TDP-43 [29]. The number of cleavages per enzyme was first divided by the total number of cleavage sites across each protein to calculate its contribution percentage. Because each protein differs in amino acid length, this percentage was then normalized to the mean contribution percentage of each enzyme across all four proteins. A Euclidean distance matrix comparing all normalized values was then calculated and an unsupervised single-link heirarchical clustering analysis was performed and visualized using R.

#### Correlation analysis of cleavage profiles

To compare the cleavage profiles for each enzyme across APP, we performed a Spearman’s rank-order correlation analysis of the P1 sites identified through MSP-MS. Correlation coefficients and *p*-values were then plotted in a heatmap using R.

## Results

### APP is enriched in the endolysosomal compartment and is cleaved by lysosomal proteases in vitro

Although APP is classically described as a cell-surface transmembrane protein, recent evidence indicates that both APP and its cleavage products are also enriched in subcellular compartments—most notably within lysosomes [32, 36]. In addition to being endocytosed from the cell-surface, APP can be directly transported from the trans-Golgi network to endolysosomal compartments, bypassing the plasma membrane [12, 14]. Such trafficking is critical not only for the amyloidogenic processing of APP into A$\beta$ [9, 10, 12, 14, 37] but also for the clearance of APP-derived byproducts [17, 25, 38].

**Fig. 1:**
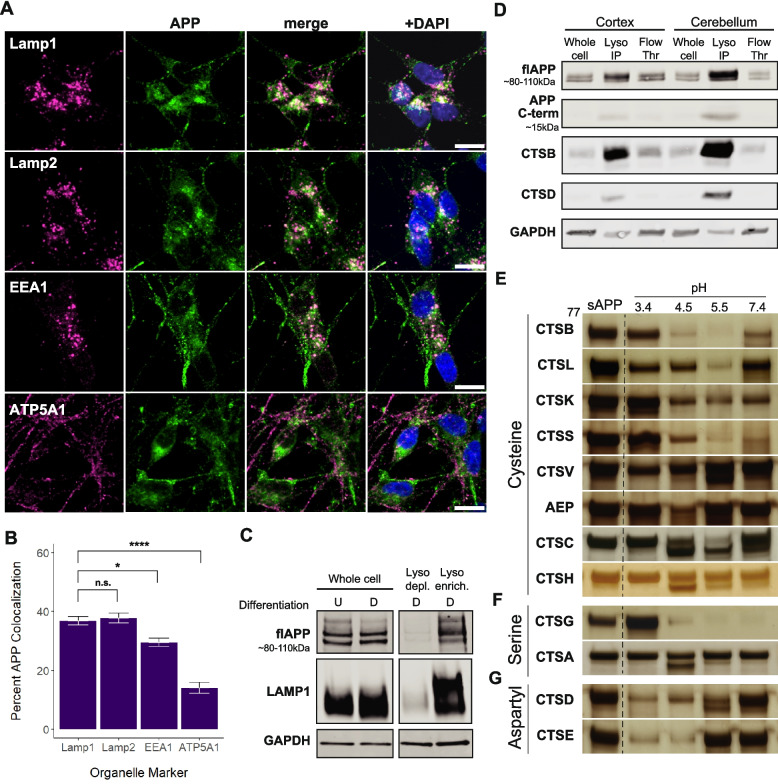
APP is enriched in the endolysosomal compartment and is cleaved by lysosomal proteases in vitro. **A** Differentiated SH-SY5Y neurons were fixed and immunostained with antibodies against organelle markers (magenta) and APP (green). Lamp1 and Lamp2, EEA1, and ATP5A1 are enriched in lysosomes, early endosomes, and mitochondria, respectively. Scale bar = 10 $\mu$m. **B** Colocalization analysis was performed as described in methods. Percent APP overlap with each organelle marker was quantified and compared using a One-Way ANOVA followed by Bonferroni post-hoc analysis. * *p* < 0.05, ** < 0.01, *** < 0.001. **C** Undifferentiated (“U”) and differentiated (“D”) SH-SY5Y cells were lysed and cellular contents were separated via density gradient centrifugation. Lysosome depleted (“Lyso depl.”) and lysosome enriched (“Lyso enrich.”) fractions were compared with whole cell samples on a Western Blot after staining with antibodies against APP, Lamp1, and GAPDH. **D** Cortex and cerebellum tissue was collected from LysoTag mice and neuronal lysosomes were collected via immunoprecipitation. Purified lysosomes (“Lyso IP”) were blotted alongside the non-immunoprecipitated flow through (“Flow Thr”) and whole cell samples. **E** Silver staining of sAPP cleaved by various lysosomal proteases and at multiple pH levels. Blots of enzymes that did not cleave sAPP are shown in Figure S4

To further define its intracellular distribution, we first examined the localization of endogenous APP in SH-SY5Y neuroblastoma cells, which can be differentiated with retinoic acid and BDNF into a neuron-like state. Using immunohistochemistry coupled with established colocalization analyses, we assessed APP overlap with markers of the lysosome (Lamp1, Lamp2), early endosome (EEA1), and mitochondria (ATP5A1). In undifferentiated SH-SY5Y cells, over half of the APP co-localized with lysosomal markers, and much less with early endosome and mitochondrial markers (Figure S1). Once differentiated into a neuron-like state, nearly 40% of endogenously expressed APP colocalized with a lysosomal markers (Fig. 1A–B). We further performed sub-cellular fractionation of differentiated SH-SY5Y cells. This confirmed that lysosome-enriched fractions contain the majority of APP (Fig. 1C). Finally, to assess APP localization in vivo, we utilized a LysoTag mouse model in which neuronal lysosomes are specifically tagged with TMEM192-HA [30] (Figure S2). Upon lysosomal immunoprecipitation (LysoIP), full-length APP was enriched in lysosomal fractions from both cortex and cerebellum (Fig. 1D, Figure S3).

Given that APP is enriched in lysosomes, we next assessed which cathepsins were capable of cleaving the lysosomal lumen-facing portion of APP, also known as soluble APP (sAPP). While previous studies have shown that CTSB, CTSD, CTSL, and CTSX can cleave APP and/or A$\beta$ [38–42], there has never been a systematic assessment of lysosomal proteases that cleave sAPP. To address this gap, we incubated recombinant sAPP with individual cathepsin proteases at pH 3.4, 4.5, 5.5, and 7.4. These conditions span the broad potential activity range of these enzymes as well as the reported range of lysosomal (3.4–5.5) and cytosolic (7.4) pH values. In doing so, we found that most cysteine proteases cleaved sAPP, albeit with variable efficiency depending upon pH (Fig. 1E, Figure S4–S5). Interestingly, CTSH exhibited cleavage against APP at pH 4.5, which was surprising given our prior work demonstrating a relative lack of activity against $\alpha$-synuclein, tau, or TDP-43 under similar conditions [29]. Notably, CTSV—which we previously found to cleave $\alpha$-synuclein, tau, and TDP-43 only at pH 3.4 [29]—exhibited a much broader activity profile for sAPP, showing cleavage at pHs 3.4, 4.5, and 5.5.

Among the serine proteases, CTSG was distinguished by robust activity across an expansive pH range (4.5, 5.5, and 7.4). CTSA also demonstrated cleavage of APP at pH 4.5, while not previously displaying any significant activity against $\alpha$-synuclein, tau, or TDP-43 in this experimental paradigm [29]. Lastly, the aspartyl proteases, CTSD and CTSE, both cleaved sAPP and favored more acidic pHs. Similar to CTSV, both CTSD and CTSE exhibited a broader range of activity on sAPP than tau, TDP-43, or $\alpha$-synuclein.

Together, these findings reveal a diverse pH-dependent landscape for cathepsin-mediated APP processing. They highlight the unique ability of APP among neurodegenerative disease-relevant substrates to be cleaved by CTSA and CTSH and underscore the importance of systematically profiling these enzymes to better understand their potential roles in Alzheimer’s disease.

### MSP-MS provides a detailed cleavage map of APP by lysosomal proteases

To obtain an even more precise view of lysosome-dependent APP proteolysis, we next employed MSP-MS to map cleavage sites across the entire sequence of APP. Although select cathepsin cleavage sites within the A$\beta$ region have been identified [39, 40], the full spectrum of lysosomal protease activity on APP has not been previously documented. To perform these experiments, we generated a custom library of overlapping 18-amino acid peptides spanning the longest human APP isoform (APP-770) and incubated this library with 13 different proteases under multiple pH conditions and time points (Table S1, Figure S6).

**Fig. 2:**
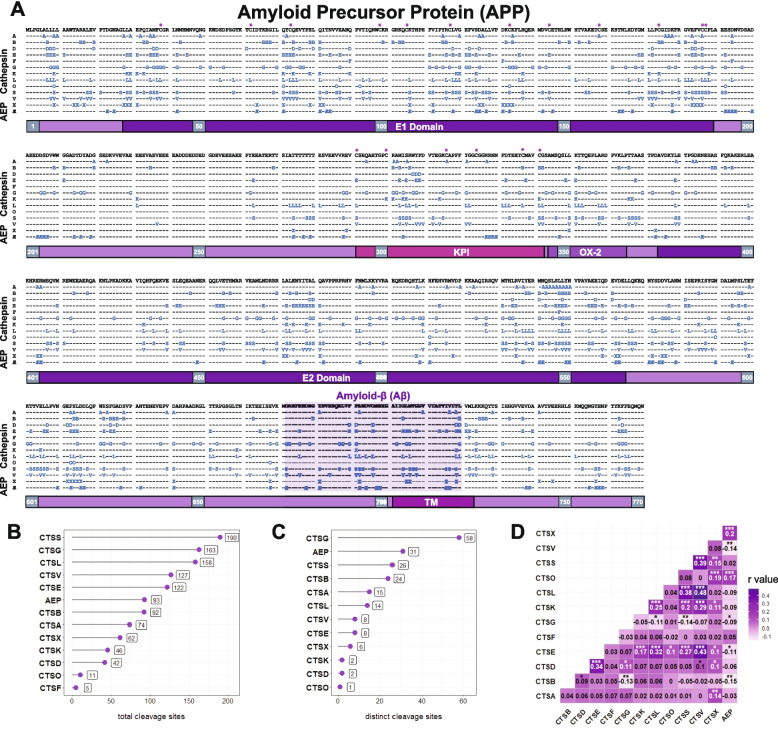
MSP-MS provides a detailed cleavage map of APP by lysosomal proteases. **A** A comprehensive cleavage map of APP generated through MSP-MS. P1 cleavage sites are indicated with a corresponding letter (i.e. CTSA = “A”), or by the symbol “Æ” for Asparagine Endopeptidase (AEP/legumain). To generate accurate spectra, cysteines were replaced by alanines as described in Methods (indicated with a ‘*’). **B** A bar graph representing the number of unique cleavage sites across the sequence of APP-770 for each enzyme. **C** Distinct cleavage sites—defined as P1 sites that were only identified for a single cathepsin—were quantified and are displayed as a bar graph. **D** A correlation plot visualizing the similarity in cleavage sites between enzymes. Darker values represent a greater degree of overlap between proteases. * *p* < 0.05, ** < 0.01, *** < 0.001

This MSP-MS approach revealed a total of 390 unique lysosomal protease cleavage sites in APP (Fig. 2A–B). The proteases we tested generated between 5 (CTSF) and 190 (CTSS) sites. We confirmed that many of the cathepsins previously shown to efficiently cleave recombinant sAPP, such as CTSS, CTSG, and CTSL, yielded the highest number of cleavage events. We also compared APP lysosomal proteolysis to that of tau, TDP-4 and a-synuclein [29]. Interestingly, while CTSL and CTSS were prominent contributors to APP cleavage (paralleling their roles in processing other neurodegeneration-related substrates), APP was generally less biased toward any one protease compared with tau, $\alpha$-synuclein, or TDP-43 [29] (Figure S7). We also observed that cleavage events were unevenly distributed along the APP sequence; the E1, KPI, and E2 domains contained many highly redundant protease cleavage sites, whereas the flexible linker between E1 and the KPI was notably resistant to cleavage (Fig. 2A).

Analysis of “hot spots” and “quiet zones” also revealed differences in cleavage redundancy. Certain regions of APP, such as the E1 domain, were targeted by many proteases, while other areas of APP, such as the region between the E1 and KPI domains, exhibited a relative paucity of cleavage sites (Fig. 2A). Among the non-redundant or “distinct” cleavage sites, CTSG stood out as representing a large proportion of these (58 of 195 total sites, or 29.7%), which was nearly twice the number of sites targeted by the next most favored enzyme, AEP (Fig. 2C). Overall, roughly half of all identified sites (195 of 390) were cleaved by a single protease, suggesting that even small shifts in protease activity or expression during disease could substantially impact APP processing.

To further assess the redundant versus non-redundant activity among these proteases, we also performed pairwise correlation analyses. We found that CTSS and CTSL, which cleave at 48.7% and 40.5% of identified cleavage sites, respectively, also showed considerable overlap with CTSE, CTSK, and CTSL (Fig. 2D). As expected, the cleavage profiles of the aspartyl proteases CTSD and CTSE were positively correlated with one another. Meanwhile, enzymes with distinctive cleavage sites, such as AEP, CTSS, and CTSA, exhibited limited overlap with others. Much like our previous study of $\alpha$-syn, tau, and TDP-43, CTSB exhibited weak or negative correlations with most other cathepsins, further indicating that its loss of function might disproportionately impair lysosomal function.

### Lysosomal proteases exhibit unique substrate specificities that are pH-sensitive

Lysosomes typically maintain an acidic pH (approximately 4.0–4.5) that is optimal for many proteases to recognize and cleave specific substrates. While it is well-known that protease efficiency is dependent on pH and that pH optima differ between enzymes, we wondered if pH changes could alter the substrate specificity of these enzymes.

**Fig. 3:**
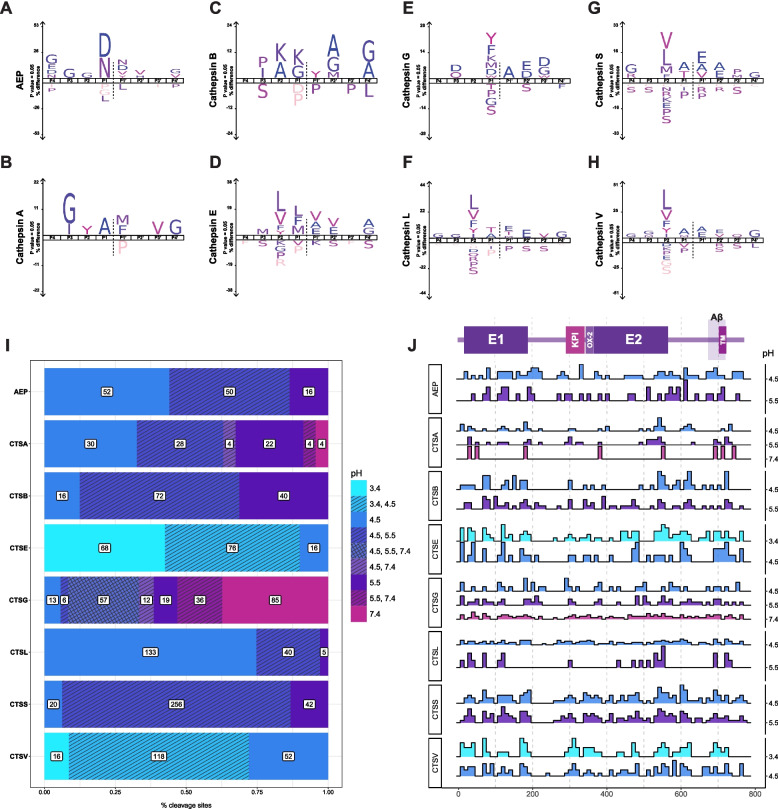
Lysosomal proteases exhibit unique substrate specificities that are pH-sensitive. **A**–**H** iceLogos generated for select enzymes based on APP MSP-MS data (additional iceLogos available in Figures S8–S9). **I** Stacked barplots representing the number of cleavage sites tallied at each pH **J** Graphical overview of the APP-770 protein and a map of cleavage patterns across APP at varying pH levels. Dotted lines occur every 100 amino acids. E1, E2 = E1 and E2 domains, respectively. KPI = Kunitz Protease Inhibitor domain. TM = transmembrane. Histograms were generated at each experimental pH value and protease combination tested using MSP-MS. Bar heights indicate the relative number of cleavage sites for each condition (ie. the area under each plot = 100%)

To this end, we first created iceLogos (i.e., probability-based visualizations of consensus cleavage site sequences) for each enzyme to assess their individual substrate specificity and compared these profiles with known motifs from the MEROPS database [43] (Fig. 3A–H, Figure S8–9). Most cysteine (CTSB, CTSL, CTSS, CTSV, CTSK, and AEP) and aspartyl proteases (CTSD and CTSE) displayed cleavage patterns consistent with established substrate preferences. However, the substrate preferences of the serine proteases (CTSA and CTSG) are less well described. As such, we integrated our APP dataset with our previous work on tau, $\alpha$-synuclein, and TDP-43 [29], to shed light on the substrate specificities of these enzymes (Figure S10). In doing so, we found that CTSA had a substrate preference for non-polar residues between P3-P3’, whereas CTSG exhibited a preference for bulky amino acids, including those with aromatic rings. By defining the specificities of CTSA and CTSG and reinforcing the profiles of the cysteine and aspartyl enzymes, we have generated a robust, comparative atlas of the cleavage motifs of the lysosomal proteases that extends beyond current database annotations.

Lysosomal pH is known to become more alkaline with age in yeast and *C. elegans* [44, 45]. To assess how age-associated pH changes can affect APP processing, we first assessed the optimal pH at which each protease cleaved APP. We found that most proteases cleaved APP most efficiently at their known pH optima, as referenced in MEROPS and the Brenda enzyme database [43, 46]. However, it was notable that a one-unit increase in pH dramatically altered the number of cleavage sites on APP for several proteases. For example, at pH 4.5, AEP cleaved 102 sites on APP, but at pH 5.5, only 66 remained. Even more dramatically, a pH increase of 4.5 to 5.5 contracted the number of CTSL cleavage sites in APP from 173 to 45, an $\sim$84% reduction (Fig. 3I–J). Given CTSL’s broad neuronal expression and central role in our cleavage map, this remarkable pH sensitivity may help explain diminished APP clearance in less acidic lysosomes—a phenomenon observed in disease contexts [20, 41, 47]. Furthermore, many of these pH-dependent changes were observed within the A$\beta$ region, underscoring their potential relevance to AD pathology.

### Disease-associated mutations alter cathepsin cleavage of A$\beta$ region

Extensive research has focused on secretase-mediated cleavage of APP and has carefully characterized the pathogenic mechanisms leading to A$\beta$ accumulation in neurodegeneration [48, 49]. For example, the AD-causing Swedish mutation (KM670/671NL) enhances $\beta$-secretase processing, leading to increased A$\beta$ production, while the London mutation (V717I) shifts $\gamma$-secretase activity to favor the more amyloidogenic A$\beta$−42 isoform [50, 51]. By contrast, the A673T variant in APP reduces $\beta$-secretase cleavage and lowers amyloid accumulation, thereby decreasing AD risk and acting as a “protective” variant [52]. These mutations highlight the importance of secretase cleavage in AD.

However, other pathogenic variants in APP have been identified that are removed from secretase cleavage sites and thus do not appear to impact secretase activity. The pathogenic mechanisms in these variants are less well understood. Examples of such mutations include the APP Arctic (E693G) and Dutch (E693Q) variants, which are associated with AD and cerebral amyloid angiopathy, respectively. Interestingly, prior work suggests that mutations at position 693 reduce A$\beta$ levels, increase A$\beta$ N-terminal fragments, and result in little to no amyloid PiB uptake in human studies [53–55]. These phenomena have been attributed to more rapid oligomerization and fibrillization of the variant fragments compared to wild-type [56–59]. In this context, we assessed our newly generated lysosomal protease cleavage map of APP and noted that ten different proteases can cleave APP at or proximal to position 693. Thus, we hypothesized that the mutant amino acid could promote lysosomal protease processing of APP, thereby contributing to the diminished levels of A$\beta$.

**Fig. 4:**
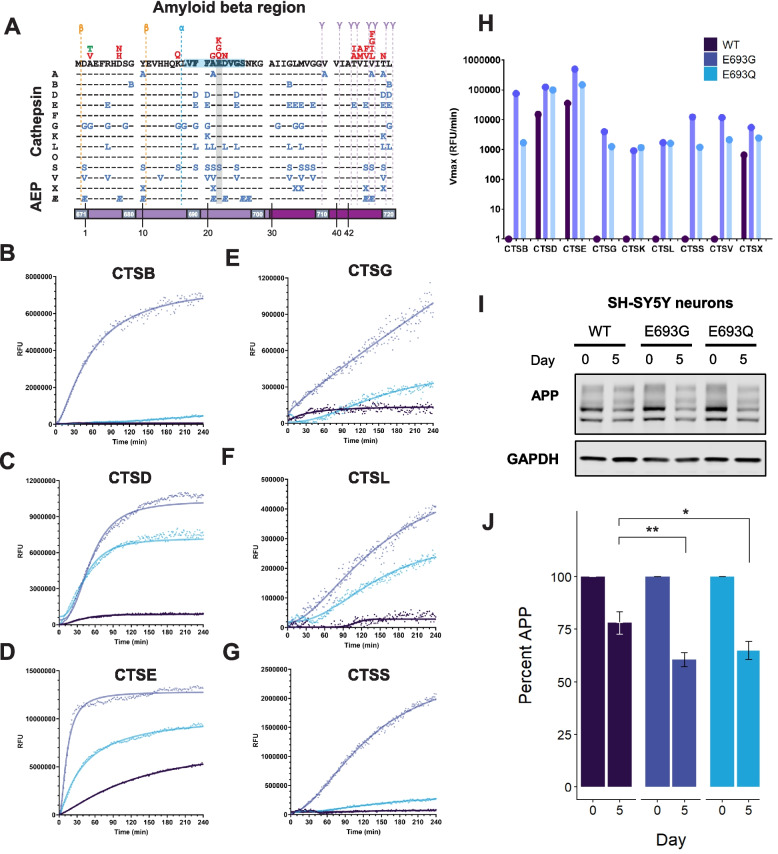
Disease-associated mutations alter cathepsin cleavage of A$\beta$ region. **A** Expanded A$\beta$ region of the MSP-MS cleavage map from Fig. 2. Dotted lines represent known $\beta$, $\alpha$, and $\gamma$ cleavage sites. Protective (green) and deleterious (red) variants are plotted above the primary sequence. A$\beta$ numbering is shown in black. Site E693 is highlighted with a grey bar and the 9 amino acid region used for peptide cleavage assays is highlighted in blue. **B**–**G** E693 variants alter cleavage by lysosomal proteases. Wild-type, Arctic (E693G), and Dutch (E693Q) fluorogenic peptides were incubated with various cathepsins. Cleavage activity was determined by quantifying fluorescence over time. **H** Maximum reaction velocity (Vmax) of the cleavage assays shown in (**B**–**G**). RFU/min are plotted on a logarithmic scale. **I** Differentiated wild-type and E693 variant SH-SY5Y cells were collected at 0 and 5 days following doxycycline induction of APP expression. **J** Band intensity from (**I**) was quantified and normalized to GAPDH on Western Blots. Error bars represent ± SEM. n = 8; * *p* < 0.05, ** < 0.01, *** < 0.001

To test this, we performed fluorescence-based in vitro protease activity assays. Peptide substrates spanning the E693 region were synthesized with either the wild-type sequence or the E693G/Q mutation and then labeled with a fluorescent moiety and a quencher. Peptides were then incubated with individual proteases. The wild-type peptide was poorly or modestly cleaved by most of the cathepsins tested. In contrast, many cathepsins cleaved the E693G and E693Q peptides more rapidly than the wild-type peptide (Fig. 4B–G). In particular, CTSB, CTSG, CTSK, CTSL, CTSS, and CTSV exhibited little or no activity against the wild-type peptide but robust cleavage of the mutant peptides (Fig. 4H). Of note, no protease displayed a reduction in activity with the mutant substrates compared to wild type (Figure S11). Thus, the Arctic and Dutch APP mutations may be “amyloid poor” disease variants because they enhance cleavage in the middle of the A$\beta$ peptide.

To validate these findings in a cellular context, we generated stable SH-SY5Y cell lines harboring inducible, FLAG-tagged APP (WT, E693G, or E693Q). Following terminal differentiation into neuron-like cells, FLAG-APP expression was induced. Lysates were collected at day 0 and day 5 post-induction, and APP levels were quantified via anti-FLAG Western blot. Over five days, APP levels in cells expressing E693G or E693Q variants decreased by an average of $\sim$40%, compared to a $\sim$25% decrease in wild-type cells (Fig. 4I–J, Figure S12). A similar trend was observed for A$\beta$−42 levels via ELISA, though the difference did not reach statistical significance (Figure S13). Notably, APP and A$\beta$−42 clearance was less robust in these cell lines compared with our in vitro experiments using E693 mutant peptides. The in vitro cleavage experiments quantify the action of a single protease over an extended period, allowing for the calculation of a rate of proteolysis (Vmax). In contrast, the cell-based assays (Fig. 4I–J) offer a snapshot of the steady state levels of APP and A$\beta$−42, reflecting the sum of proteolytic cleavage events by all lysosomal proteases. Together, these observations show that E693G and E693Q enhance their cleavage by lysosomal proteases compared to wild-type APP, an effect that is observable in cells and which would likely be compounded over an extended period in vitro.

### APP augments Tau cleavage by CTSG

Amyloid plaque deposition precedes tau tangle pathology by many years, yet a direct causative link between APP biology and tau accumulation remains elusive. Prior studies have examined the potential toxic gain-of-function of amyloid oligomers and aggregates, as well as potential loss-of-function mechanisms [60–62]. In examining the interplay of APP with lysosomal proteases, we noted that sAPP harbors a serine protease inhibitor domain known as the KPI domain. In fact, the KPI domain is such a potent inhibitor of the serine proteases thrombin and trypsin that APP is also known as Protease Nexin II [63]. We therefore wondered if the sAPP portion of APP might inhibit degradation of tau by lysosomal serine proteases.

**Fig. 5:**
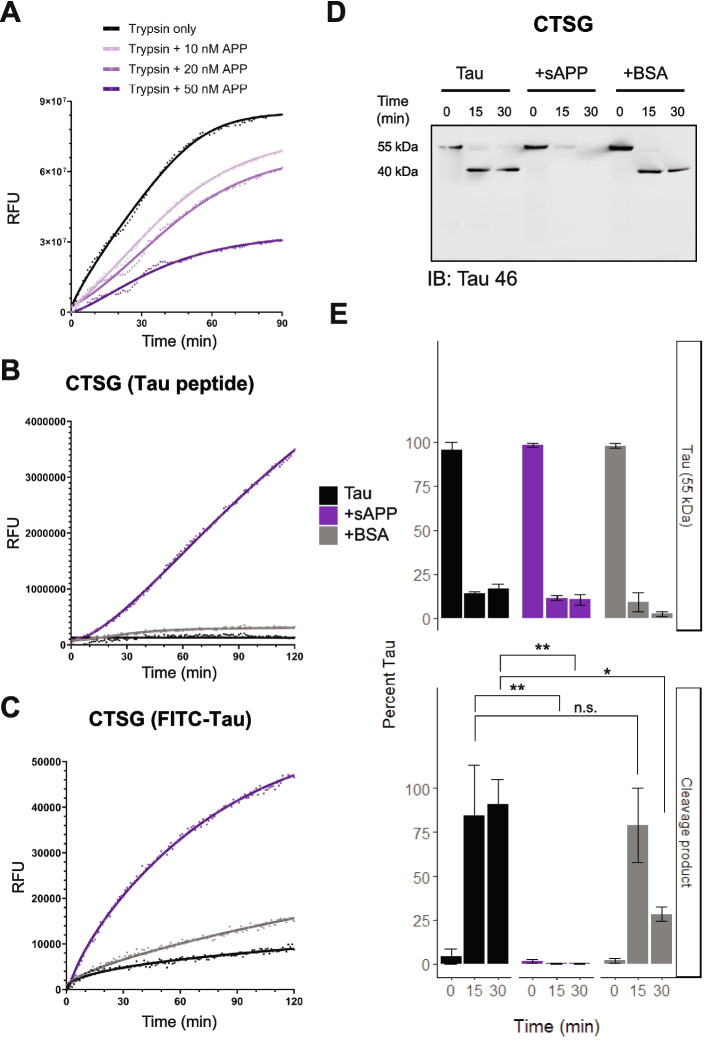
APP augments Tau cleavage by CTSG. **A** Activity assay measuring cleavage of a fluorogenic APP peptide in the presence of increasing Trypsin concentrations. **B**–**C** Activity assays measuring the cleavage of a fluorogenic tau-peptide (**B**) and FITC-labelled tau (**C**) by CTSG over time in the presence of either sAPP or BSA. **D** Recombinant human tau-441 was cleaved in vitro by CTSG in the presence of either sAPP or BSA and analyzed via Western Blot. **E** Quantification of (**D**), measuring tau (55 kDa) as well as a prominent cleavage product (40 kDa). A two-way repeated measures ANOVA followed by Holm-Bonferroni correction post-hoc correction. Error bars represent ± SEM. n = 3; * *p* < 0.05, ** < 0.01, *** < 0.001

To test this, we first confirmed that sAPP indeed inhibits trypsin in a dose-dependent manner (Fig. 5A, Figure S14A). We next tested the ability of sAPP to influence the cleavage of a tau peptide by the lysosomal serine protease, CTSG at pH 4.5. This peptide, spanning amino acids 274–282 and includes a P1 CTSG cleavage site at amino acid 280 [29]. To our surprise, sAPP did not inhibit CTSG cleavage of tau. Rather, sAPP dramatically increased the rate of CTSG cleavage of this tau peptide (Fig. 5B). Addition of sAPP also accelerated the proteolysis of full-length FITC-labeled tau by CTSG (Fig. 5C, Figure S14B). Interestingly, this phenomenon seemed specific to tau, as sAPP did not alter CTSG cleavage of the casein-based, “universal” substrate (Figure S15).

Finally, we measured the proteolysis of full-length, unlabeled tau by CTSG without or with sAPP. The addition of sAPP greatly accelerated the cleavage of tau by CTSG (Fig. 5D, Figure S16). Within the same time frame, CTSG alone or with BSA (as a control for molecular crowding), produced only partial tau cleavage and led to the emergence of a prominent 40 kDa fragment. CTSL, a cysteine protease, did not show the same acceleration of tau cleavage by sAPP, compared to CTSG (Figure S17). The ability of sAPP to substantially augment CTSG cleavage of tau suggests that sAPP may serve a neuroprotective role, facilitating the lysosomal clearance of tau.

## Discussion

This study provides the most in-depth characterization of lysosomal protease-mediated degradation of APP to date, revealing novel insights into the pH-dependence, mutation-sensitivity, and broader implications of this process for AD. Our findings complement existing models of APP processing, highlighting the lysosome as a critical, yet underappreciated, site of APP metabolism and a potential therapeutic target.

We first confirmed that APP is significantly enriched within the endolysosomal system of both neuronal cell lines and mouse brain tissue, consistent with previous reports [12, 13, 17, 64]. This localization is crucial, as it places APP in direct contact with the lysosomal proteases responsible for its degradation. Our in vitro assays demonstrate that a wide range of cathepsins, including cysteine (CTSB, CTSC, CTSH, CTSK, CTSL, CTSS, CTSV, AEP), serine (CTSA, CTSG), and aspartyl (CTSD, CTSE) proteases, can cleave sAPP. This broad susceptibility contrasts with the more selective cleavage patterns we previously observed for other neurodegeneration-related proteins like tau, $\alpha$-synuclein, and TDP-43 [29]. The finding that CTSH, CTSV, and CTSA cleave APP, but not the other tested substrates, highlights a unique aspect of APP’s lysosomal processing.

Next, our MSP-MS analysis provides, to our knowledge, the first comprehensive map of lysosomal protease cleavage sites across the full-length APP-770 isoform. This map reveals a complex landscape of proteolytic susceptibility, with “hot spots” of high cleavage activity in the E1, KPI, and E2 domains, and a relatively resistant flexible linker region. The identification of both redundant cleavage sites (targeted by multiple proteases) and distinct sites (specific to a single protease) suggests that shifts in lysosomal protease expression or activity, which can occur during aging and disease [18, 23, 65], could significantly alter APP processing and potentially contribute to AD pathogenesis.

Critically, this study demonstrates the pH-dependence of cathepsin-mediated APP cleavage. While most proteases exhibited activity profiles against APP that are consistent with their known pH optima, we observed a particularly striking sensitivity for certain cathepsins, such as CTSL, with activity drastically reduced by even small pH increases. Given the established role of lysosomal dysfunction in AD and other neurodegenerative diseases [20, 47, 66, 67], this finding suggests that even subtle changes in lysosomal pH could significantly alter or impair APP clearance. Such a phenomenon could potentially occur with aging, physiological stress or neurodegenerative disease, potentially contributing to the accumulation of APP fragments and exacerbating disease progression.

Our investigation into the Arctic (E693G) and Dutch (E693Q) APP mutations revealed a surprising and potentially significant finding: these mutations enhanced cleavage by several key lysosomal proteases, including CTSD, CTSL, and CTSG and suggests that A$\beta$ has a more complicated relationship with protease activity than was previously appreciated. At the very least, these findings seemingly clarify why the Arctic and Dutch mutations lead to amyloid poor forms of disease.

Perhaps our most surprising finding is the dramatic enhancement of tau proteolysis by CTSG in the presence of sAPP. This observation unveils a previously unrecognized interaction between APP and tau within the lysosomal environment. CTSG is a unique cathepsin and has long been of interest in understanding various forms of dementia. Unlike many other cathepsins, it is active at neutral pH and can be found extracellularly. CTSG is a marker of reactive astrocytes in the brain [68] and is differentially expressed in patients with age-associated cognitive decline and Lewey Body Dementia [69–71].

Although sAPP is a well-established serine protease inhibitor, its *augmentation* of CTSG’s activity on tau, rather than inhibition, is counterintuitive. This suggests a complex underlying mechanism, potentially involving allosteric modulation of CTSG, altered substrate presentation, or the formation of a ternary complex comprising sAPP, tau, and CTSG. Such a facilitating role within the lysosome is not without precedent; for instance, progranulin has been shown to facilitate the maturation of CTSD, thereby enhancing its proteolytic activity [72]. Similarly, saposin C is known to enhance the activity of glucocerebrosidase on glucosylceramide [73]. Further investigations are currently underway to elucidate the precise molecular mechanism of this newly described lysosomal function of sAPP.

This finding carries significant implications for understanding the intricate interplay between APP and tau pathologies in AD. It raises the compelling possibility that APP, beyond its role as the source of A$\beta$, may directly influence tau clearance within the lysosome. Consequently, a loss of this sAPP-mediated enhancement of tau degradation could directly contribute to the accumulation of pathological tau, thus offering a novel perspective on AD progression.

Acknowledging the inherent challenges in fully replicating physiological complexity, this study does possess certain limitations. While our in vitro assays offer valuable, detailed insights into protease activity, they cannot entirely recapitulate the intricate environment of a living cell. Future research will therefore extend to more complex cellular models, including primary neurons and induced pluripotent stem cell (iPSC)-derived neurons from patients carrying APP variants, to validate these findings in a more biologically relevant context. We observed for example that the effects of E693 variants on APP cleavage were less dramatic in differentiated SH-SY5Y neurons than in vitro assays. Given that SH-SY5Y neurons are relatively immature, it would be interesting to examine this phenomenon in cells that better recapitulate aging neurons, which have a reduced capacity for maintaining protein homeostasis.

Limitations to MSP-MS cleavage mapping should also be acknowledged. While this technique provides a comprehensive profile of possible cleavage sites across APP, the likelihood of each cleavage event will depend on the availability of various proteases, the intracellular environment, and lysosomal pH, as we have identified here. Additionally, APP conformation, post-translational modification, the presence of cysteines forming disulfide bonds, and other factors will likely alter cleavage activity in vivo. Although the MSP-MS map will be useful for generating hypotheses regarding APP proteolysis, cleavage sites of interest should be confirmed using parallel methods. Furthermore, while our LysoTag mouse model served as a valuable tool for investigating lysosomal APP processing in vivo, additional studies are necessary to confirm these observations in human brain tissue, an area that has seen limited extensive past exploration [74]. Finally, the exact molecular mechanism by which sAPP enhances CTSG-mediated tau degradation warrants comprehensive investigation.

## Conclusion

Our study provides compelling evidence that lysosomal protease-mediated degradation of APP is a complex, pH-regulated, and mutation-sensitive process with significant implications for AD pathogenesis. The detailed cleavage map generated by MSP-MS, the unexpected effects of the Arctic and Dutch mutations, and the novel discovery of sAPP’s role in augmenting tau degradation by CTSG, all highlight the importance of considering the lysosome as a central hub in the interplay between APP and tau pathologies. These findings open new avenues for research and suggest that therapeutic strategies targeting lysosomal function, specifically modulating cathepsin activity or enhancing sAPP’s interaction with tau and CTSG, may hold promise for treating AD and related neurodegenerative diseases.

## Additional file

Additional file 1. Additional file 1: Figure S1-S17; Table S1-3; Supplemental Data

## Data Availability

All data generated or analyzed during this study are either included in this published article (Supplemental Data) or are available from the corresponding author on reasonable request. An interactive protein visualization tool is available online for APP MSP-MS data at https://apps.rtemis.org/rtemisseq/.

## Abbreviations

APP: Amyloid Precursor Protein
A$$\beta$$: amyloid beta
sAPP: soluble APP
AD: Alzheimer’s disease
MSP-MS: Multiplexed Substrate Profiling by Mass Spectrometery
CTSA: Cathepsin A
CTSB: Cathepsin B
CTSC: Cathepsin C
CTSD: Cathepsin D
CTSE: Cathepsin E
CTSF: Cathepsin F
CTSG: Cathepsin G
CTSH: Cathepsin H
CTSK: Cathepsin K
CTSL: Cathepsin L
CTSO: Cathepsin O
CTSS: Cathepsin S
CTSV: Cathepsin V
CTSX: Cathepsin X
AEP: Asparagine endopeptidase
ACH: Amyloid Cascade Hypothesis
KPI: Kunitz Protease Inhibitor domain
