# Supplementary material for: Lysosomal protease-mediated APP degradation is pH-dependent, mutation-sensitive, and facilitates tau proteolysis
Source: Mol Neurodegener Adv. 2026 Jan 19;2(1):10. doi: 10.1186/s44477-025-00017-6 (PMC12886278; doi:10.1186/s44477-025-00017-6)
Supplement: Supplementary file 1 — Additional file 1. Additional file 1: Figure S1-S17; Table S1-3; Supplemental Data [file 44477_2025_17_MOESM1_ESM.zip › Supplemental Tables - Ackley et al.pdf]

Table S1

Library Name	Protein Segment Identifier	Protein Sequence	Library Name	Protein Segment Identifier	Protein Sequence
AP_01	APP_1-18	MLPGLALLLLAAWTARAL	AP_33	APP_352-369	TTQEPLARDPVKLPTTAA
AP_02	APP_14-31	TARALEVPTDGNAGLLAE	AP_34	APP_365-382	PTTAASTPDAVDKYLETP
AP_03	APP_27-44_C38A	GLLAEPQIAMFAGRLNMH	AP_35	APP_378-395	YLETPGDENEHAHFQKAK
AP_04	APP_40-57	RLNMHMNVQNGKWDSDPS	AP_36	APP_391-408	FQKAKERLEAKHRERMSQ
AP_05	APP_53-70_C62A	DSDPSGTKTAIDTKEGIL	AP_37	APP_404-421	ERMSQVMREWEEAERQAK
AP_06	APP_66-83_C73A	KEGILQYAQEVYPELQIT	AP_38	APP_417-434	ERQAKNLPKADKKAVIQH
AP_07	APP_79-96	ELQITNVVEANQPVTIQN	AP_39	APP_430-447	AVIQHFQEKVESLEQEAA
AP_08	APP_92-109_CC98AA	VTIQNWAKRGRKQAKTHP	AP_40	APP_443-460	EQEAANERQQLVETHMAR
AP_09	APP_105-122_CC105AA	AKTHPHFVIPYRALVGEF	AP_41	APP_456-473	THMARVEAMLNDRRRLAL
AP_10	APP_118-135_C133A	LVGEFVSDALLVPDKAKF	AP_42	APP_469-486	RRLALENYITALQAVPPR
AP_11	APP_131-148_CC133AA	DKAKFLHQERMDVAETHL	AP_43	APP_482-499	AVPPRPRHVFNMLKKYVR
AP_12	APP_144-161_CC144AA	AETHLHWHTVAKETASEK	AP_44	APP_495-512	KKYVRAEQKDRQHTLKHF
AP_13	APP_157-174_C174A	TASEKSTNLHDYGMLLPA	AP_45	APP_508-525	TLKHFEHVRMVDPKKAAQ
AP_14	APP_170-187_CCC174AAA	MLLPAGIDKFRGVEFVAA	AP_46	APP_521-538	KKAAQIRSQVMTHLRVIY
AP_15	APP_183-196_CC186AA	EFVAAPLAEESDNV	AP_47	APP_534-564	LRVIYERMNQSLSLLYNV
AP_16	APP_192-203	ESDNVDSADAEE	AP_48	APP_547-564	LLYNVPAVAEEIQDEVDE
AP_17	APP_199-212	ADAEEDDSDVWWGG	AP_49	APP_560-577	DEVDELLQKEQNYSDDVL
AP_18	APP_208-223	VWWGGADTDYADGSED	AP_50	APP_573-590	SDDVLANMISEPRISYGN
AP_19	APP_219-231	DGSEDKVVEVAEE	AP_51	APP_586-603	ISYGNDALMPSLTETKTT
AP_20	APP_228-238	VAEEEEVAEVE	AP_52	APP_599-616	ETKTTVELLPVNGEFSLD
AP_21	APP_234-243	VAEVEEEEAD	AP_53	APP_612-629	EFSLDDLQPWHSFGADSV
AP_22	APP_241-247	EADDDED	AP_54	APP_625-642	GADSVPANTENEVEPVDA
AP_23	APP_245-251	DEDDEDG	AP_55	APP_638-655	EPVDARPAADRGLTTRPG
AP_24	APP_249-256	EDGDEVEE	AP_56	APP_651-668	TTRPGSGLTNIKTEEISE
AP_25	APP_254-263	VEEEAEEPYE	AP_57	APP_664-681	EEISEVKMDAEFRHDSGY
AP_26	APP_261-278	PYEEATERTTSIATTTTT	AP_58	APP_677-694	HDSGYEVHHQKLVFFAED
AP_27	APP_274-291_C291A	TTTTTTTESVEEVVREVA	AP_59	APP_690-707	FFAEDVGSNKGAIIGLMV
AP_28	APP_287-304_CC291AA_BPTI	VREVASEQAETGPARAMI	AP_60	APP_703-720	IGLMVGGVVIATVIVITL
AP_29	APP_300-317_CC316AA	ARAMISRWYFDVTEGKAA	AP_61	APP_716-733	IVITLVMLKKKQYTSIHH
AP_30	APP_313-330_CC316AA	EGKAAPFFYGGAGGNRNN	AP_62	APP_729-746	TSIHHGVVEVDAAVTPEE
AP_31	APP_326-343_CC337AA	GNRNNFDTEEYAMAVAGS	AP_63	APP_742-759	VTPEERHLSKMQQNGYEN
AP_32	APP_339-356_C341A	AVAGSAMSQSLLKTTQEP	AP_64	APP_755-770	NGYENPTYKFFEQMQN

Table S2

Cathepsins	Pre-activation:	Protease concentrations and pHs used for mass spectrometry:	Mass spectrometry time points:
CTSA	Trans-activated with CTSL following the manufacturer's recommendation. The irreversible cysteine protease inhibitor E-64 was then added for an additional 30 min at 37 °C to allow for inhibition of CTSL.	40 nM in pH 4.5 and 5.5	30min, 1h, 4h
CTSB	N/A	20 nM in pH 4.5 and 5.5	1h, 4h
CTSC	Trans-activated with CTSL following the manufacturer's recommendation.The irreversible cysteine protease inhibitor E-64 was then added for an additional 30 min at 37 °C to allow for inhibition of CTSL.	N/A	1h, 4h
CTSD	N/A	10 nM in pH 3.4 and 4.5	1h, 4h
CTSE	N/A	10 nM in pH 3.4 and 4.5	1h, 4h
CTSF	N/A	50 nM in pH 4.5 buffer	2h, 6h
CTSG	N/A	2 nM in pH 4.5, 5.5 and 7.5	30 min, 1h, 4h
CSTH	Trans-activated with thermolysin following the manufacturer's recommendation. Phosphoramidon was then added to these preparations for an additional 30 min at 37 °C to allow for inhibition of thermolysin	N/A	1h, 4h
CTSK	N/A	1 nM at pH 4.5	15min, 2h
CTSL	N/A	8.0 nM at pH 4.5 and 5.5	1h, 4h
CTSO	N/A	25 nM at pH 5.5	1h, 4h
CTSS	N/A	10 nM at pH 4.5 and 5.5	1h, 4h
CTSV	N/A	20 nM at pH 3.4 and 4.5	1h, 4h
CTSX	Preincubated with dithiothreitol (DTT) following the manufacturer's recommendation.	50 nM at pH 3.4 and 4.5	1h, 4h
AEP	N/A	20 nM at pH 4.5 and 5.5	1h, 4h

Table S3

	APP
**WT APP: 598-696**	{D-ARG}{D-ARG}{LYS(MCA)}LVFFA**E**DVG{LYS(DNP)}
**E693G**	{D-ARG}{D-ARG}{LYS(MCA)}LVFFA**G**DVG{LYS(DNP)}
**E693Q**	{D-ARG}{D-ARG}{LYS(MCA)}LVFFA**Q**DVG{LYS(DNP)}

	Tau Peptide
**WT Tau: 274-282**	{D-ARG}{D-ARG}{LYS(MCA)}KVQIINKKL{LYS(DNP)}
