# Supplementary material for: Lysosomal protease-mediated APP degradation is pH-dependent, mutation-sensitive, and facilitates tau proteolysis
Source: Mol Neurodegener Adv. 2026 Jan 19;2(1):10. doi: 10.1186/s44477-025-00017-6 (PMC12886278; doi:10.1186/s44477-025-00017-6)
Supplement: Supplementary file 1 — Additional file 1. Additional file 1: Figure S1-S17; Table S1-3; Supplemental Data [file 44477_2025_17_MOESM1_ESM.zip › Supplemental Figures - Ackley et al.pdf]

Figure S1

A. Undifferentiated SH-SY5Y cells

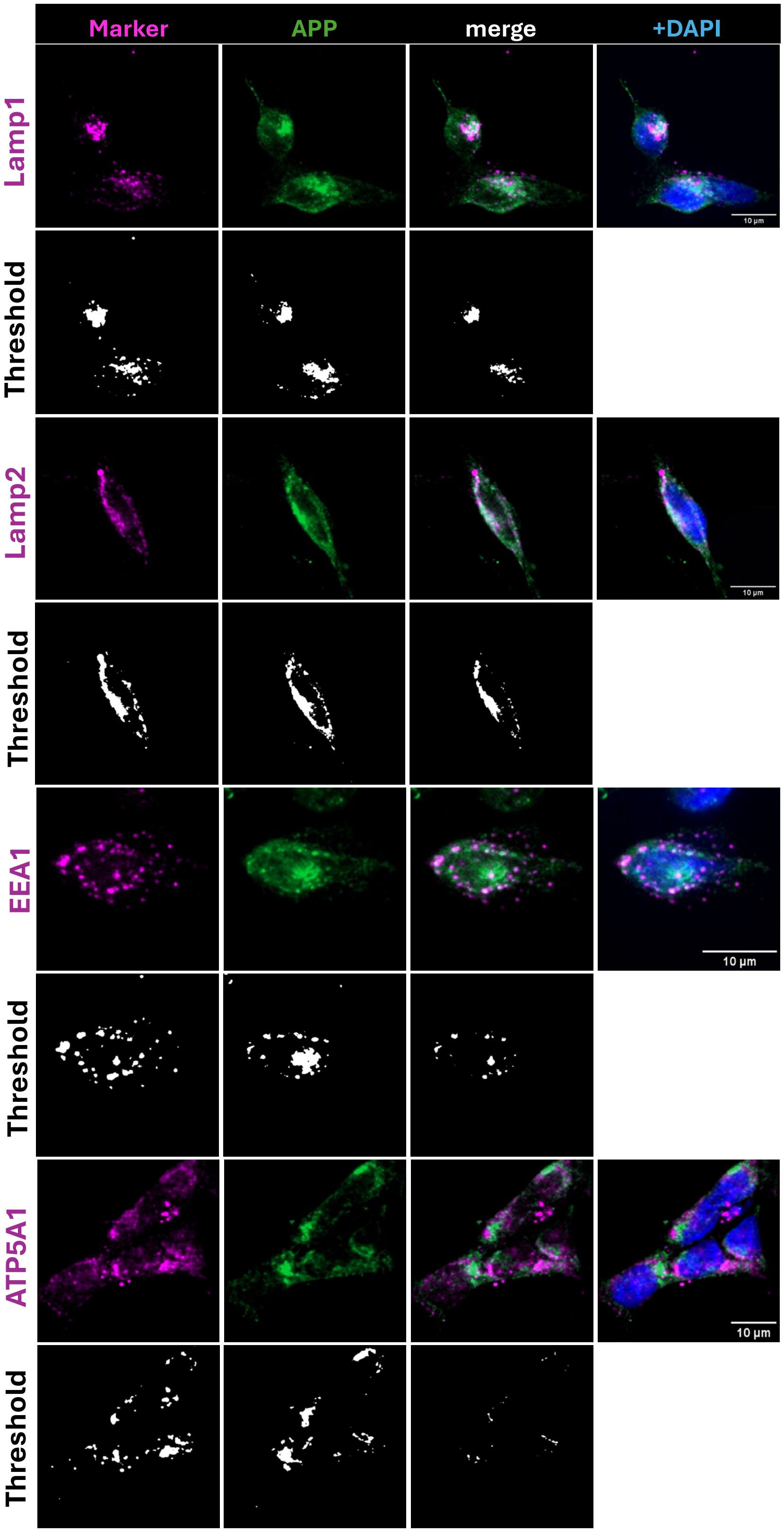

Figure S1

B. Example colocalization analysis

Organelle Marker	Area (μm^2)			% APP colocalized with Marker (Overlap / APP)
	Marker	APP	Overlap	
Lamp1	32.883	32.954	17.254	52.4%
Lamp2	31.565	32.724	19.27	58.9%
EEA1	12.156	12.345	3.313	26.8%
ATP5A1	33.695	34.954	3.125	8.9%

Figure S1. Colocalization analysis, undifferentiated SH-SY5Y neuroblastoma cells.
(A) Undifferentiated SH-SY5Y cells were fixed and immunostained with antibodies against organelle markers (magenta) and APP (green). Lamp1 and Lamp2, EEA1, and ATP5A1 are enriched in lysosomes, early endosomes, and mitochondria, respectively. Scale bar = 10 μm. For each cell, a threshold of the brightest 2% of pixels was set to create a binary mask of APP and organelle intensity. The resulting areas were then compared to find areas of overlap. **(B)** Each area was measured in μm^2, and the percentage of APP localized to the organelle in question was calculated by dividing APP area by overlap area. Example calculations are provided above, with the caveat that each cell was calculated individually and not as clusters.

Figure S2

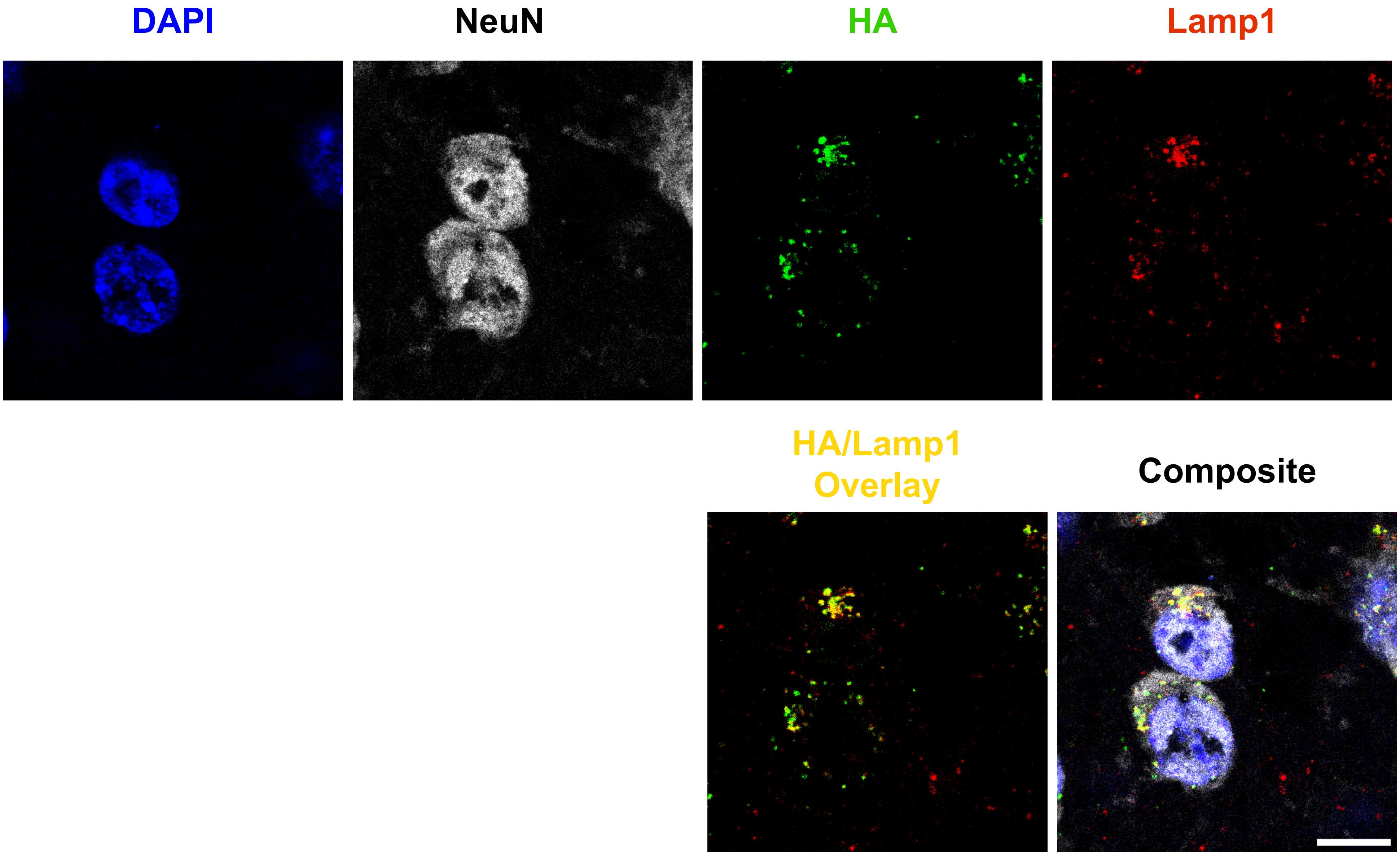

Figure S2. Validation of a neuron-specific LysoTag mouse model.
To generate a neuron-specific LysoTag mouse line, mice expressing TMEM192 fused to a 3X HA tag following a loxP STOP cassette were crossed with Syn1-Cre mice. Neuronal lysosome-specific HA expression in heterozygous mice was confirmed via immunohistochemistry and confocal microscopy. Scale bar = 10 μm.

Figure S3

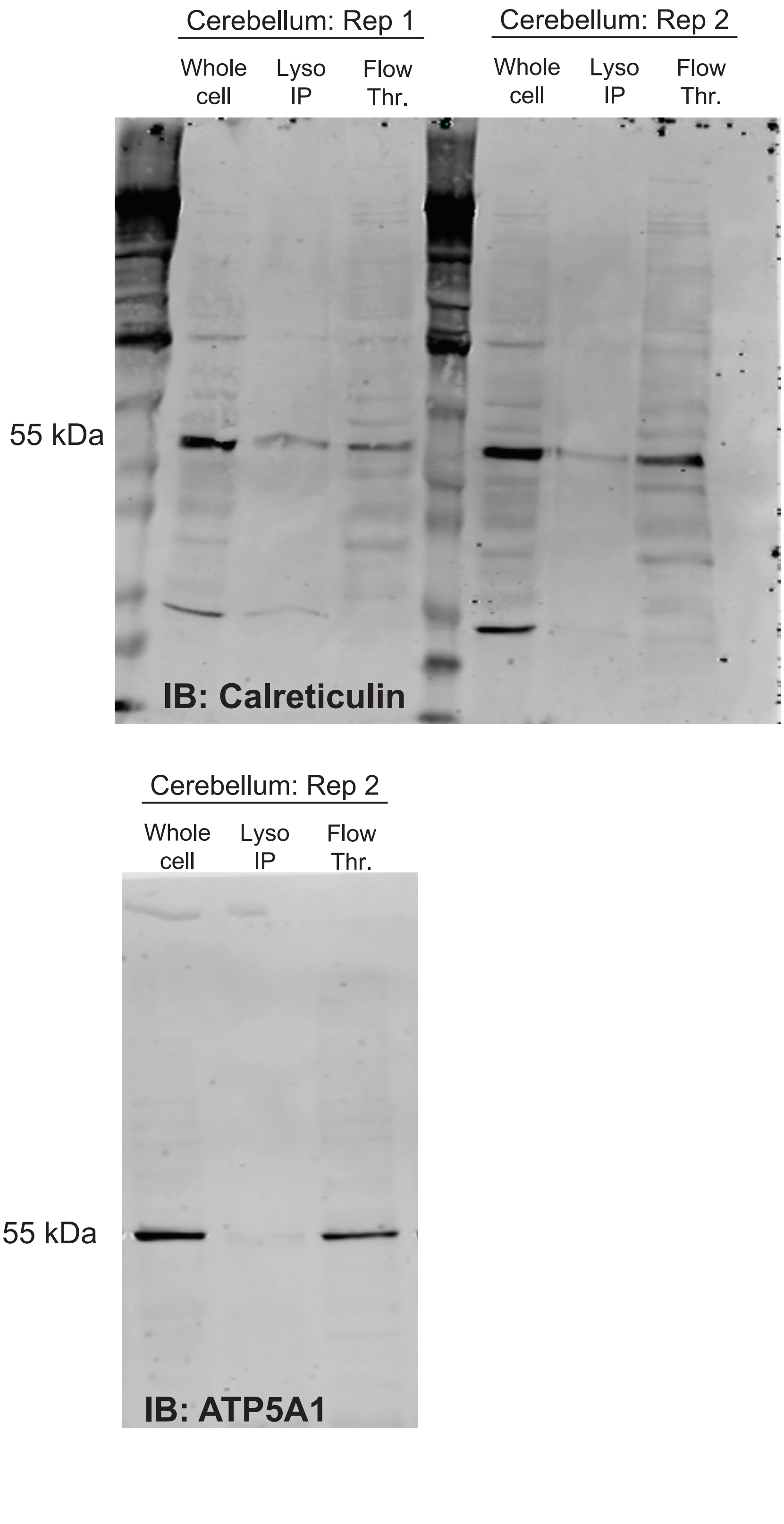

Figure S3. Additional blots testing organelle markers following mouse LysoIP.
(A) Cerebellum tissue was collected from additional LysoTag mouse replicates and neuronal lysosomes were collected via immunoprecipitation. Purified lysosomes ("Lyso IP") were blotted alongside the non-immunoprecipitated flow through ("Flow Thr.") and whole cell samples. “Rep” = replicate.

Figure S4

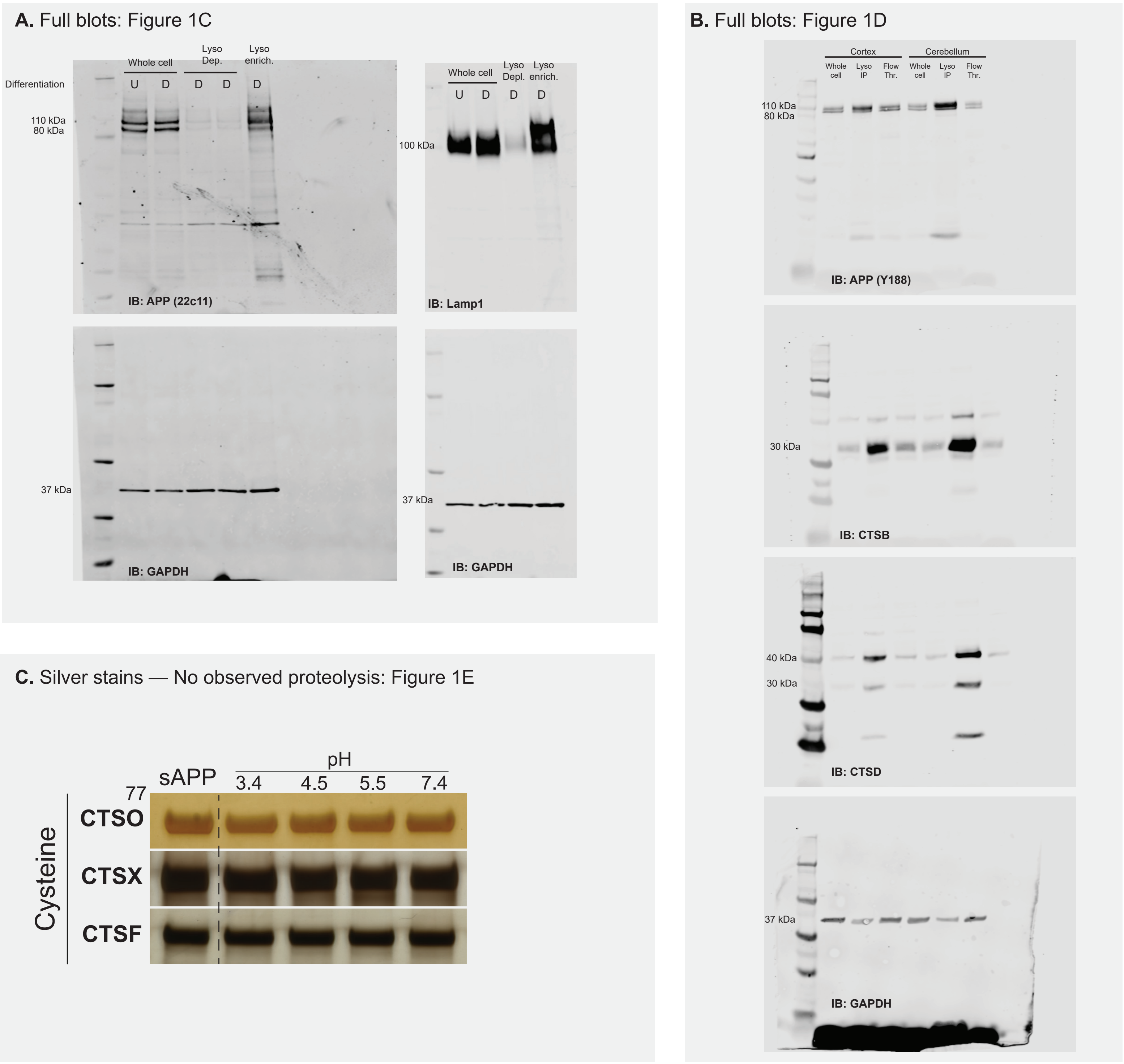

Figure S4. Full blots for Figure 1 and silver stains showing no observed proteolysis of APP by cathepsins O, X, and F.
(A) SH-SY5Y cells were lysed and cellular contents were separated via density gradient centrifugation. Lysosome depleted ("Lyso depl.") and lysosome enriched ("Lyso enrich.") fractions were compared with whole cell samples on a Western Blot after staining with antibodies against APP, Lamp1, and GAPDH. **(B)** Cortex and cerebellum tissue was collected from LysoTag mice and neuronal lysosomes were collected via immunoprecipitation. Purified lysosomes ("Lyso IP") were blotted alongside the non-immunoprecipitated flow through ("Flow Thr.") and whole cell samples.

Figure S5

Cathepsin A cleaves APP

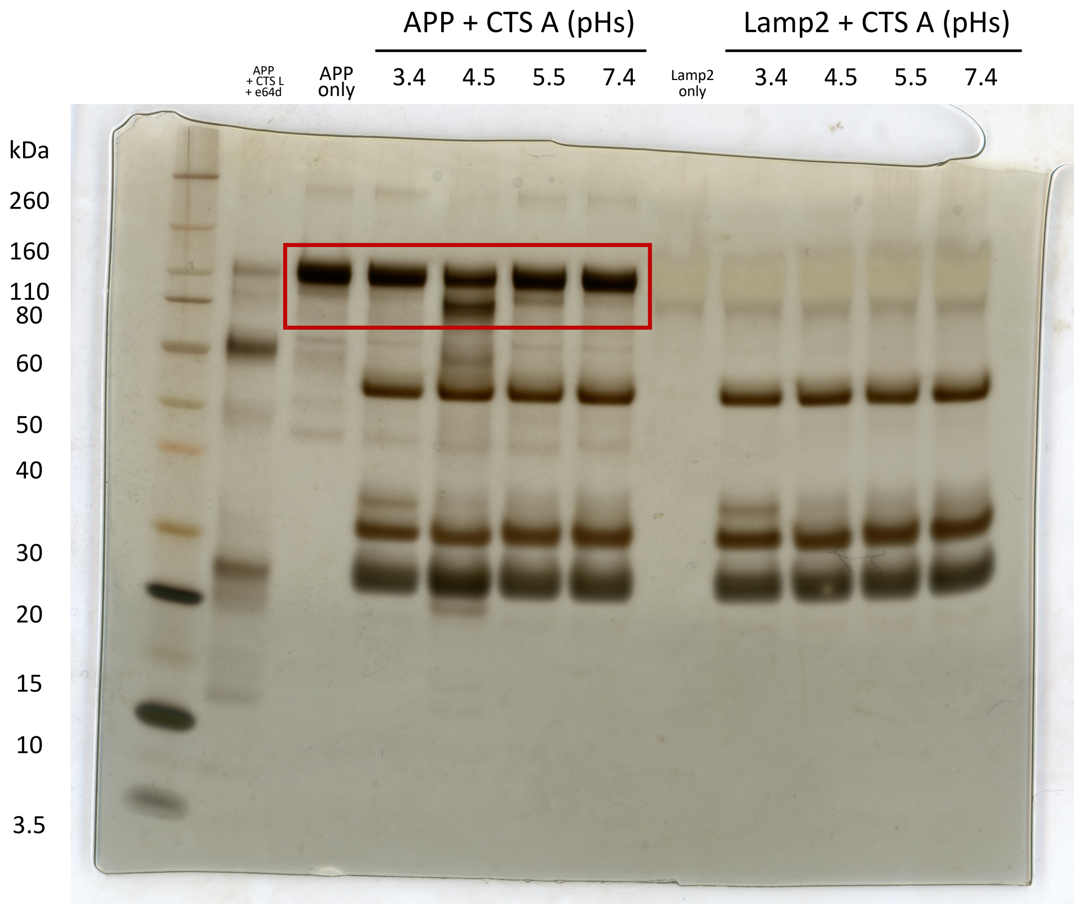

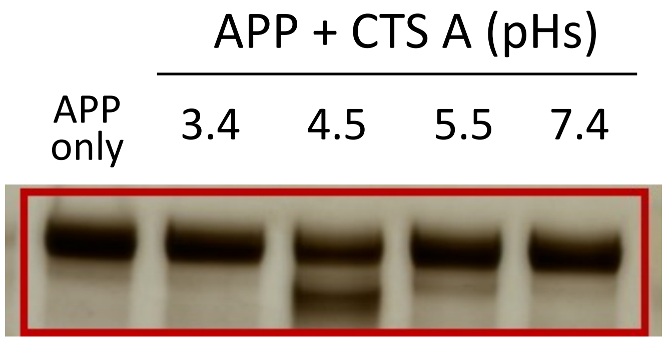

APP: 77 kDa
CTS A: 53 kDa
Lamp2: 110 kDa

Enzymes (1 uM) plus substrate (1ug) incubated at 37C for 1 hour.
Samples run on 4-12% Bis-Tris Gels with MES Buffer and Silver Stained.

Cathepsin B cleaves APP

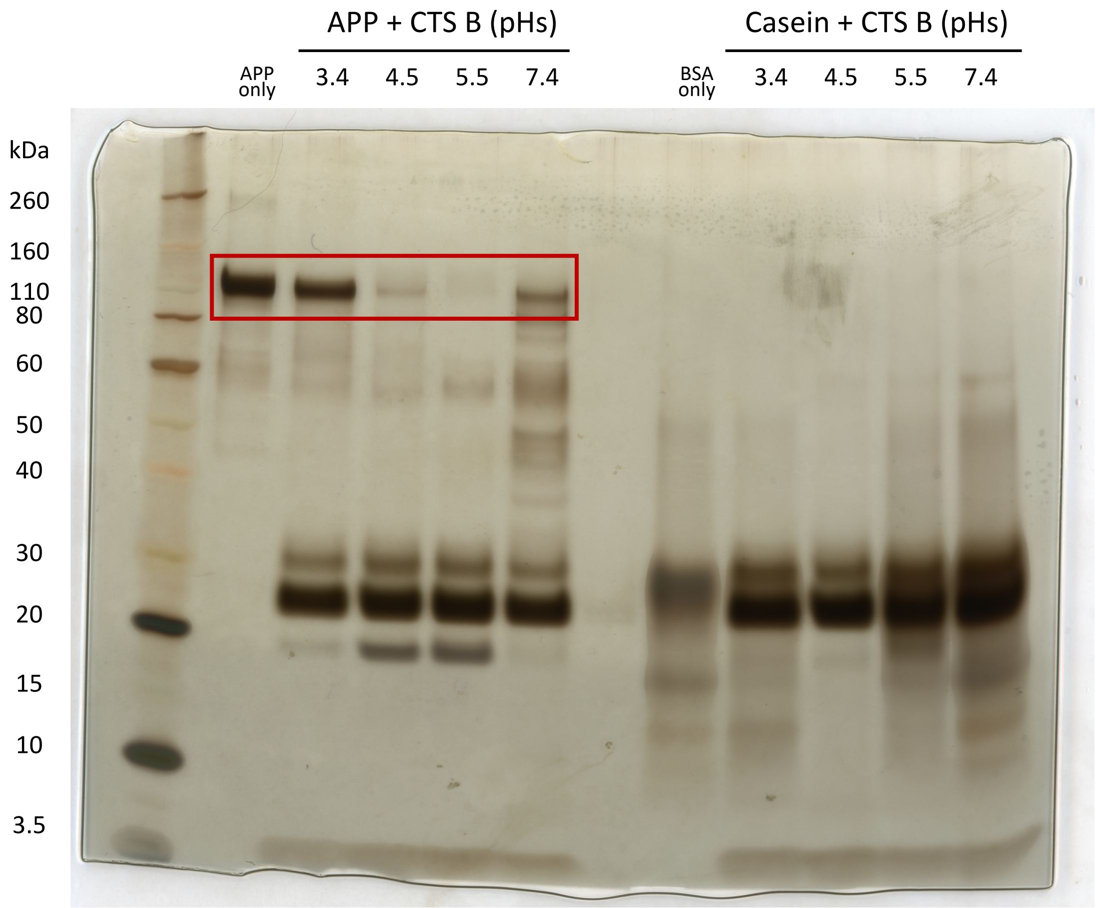

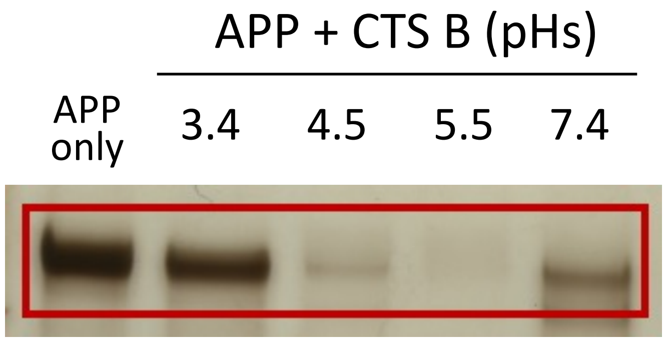

APP: 77 kDa
CTS B: 27.5 kDa
Casein: 24 kDa

Enzymes (1 uM) plus substrate (1ug) incubated at 37C for 1 hour.
Samples run on 4-12% Bis-Tris Gels with MES Buffer and Silver Stained.

Cathepsin C cleaves APP

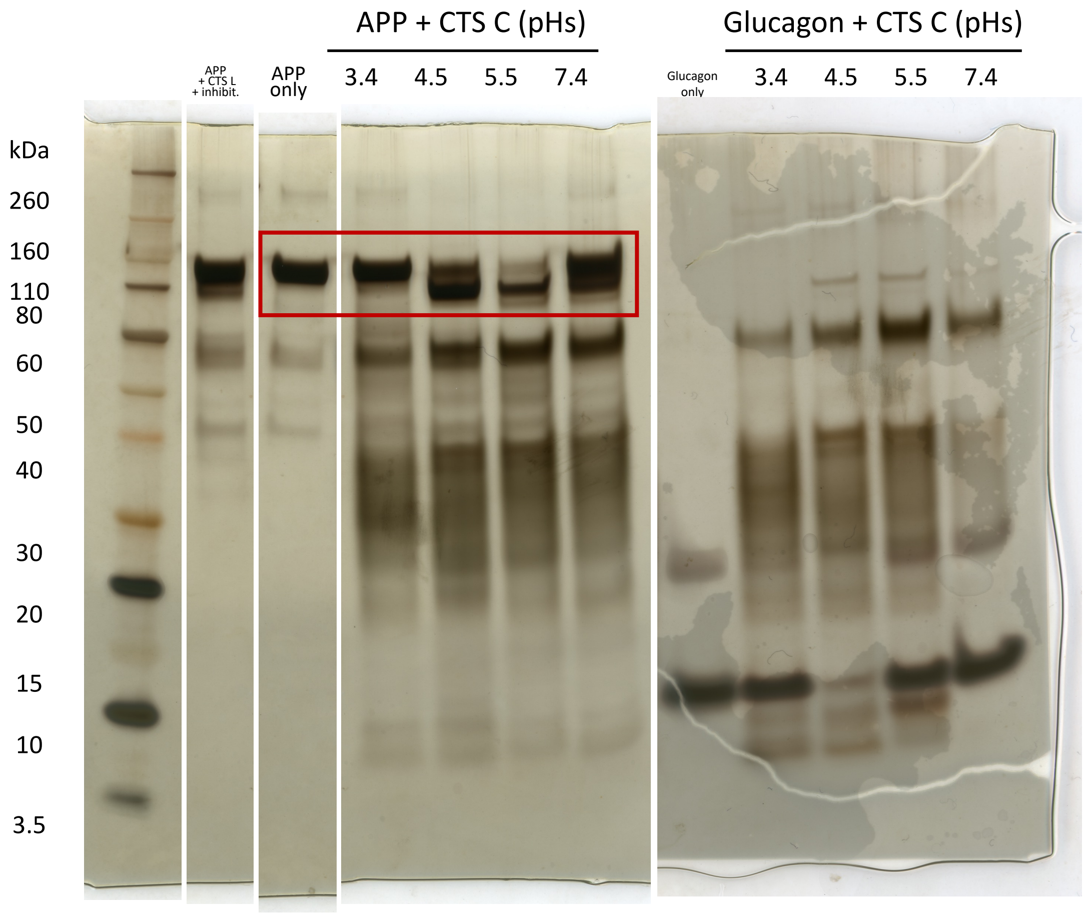

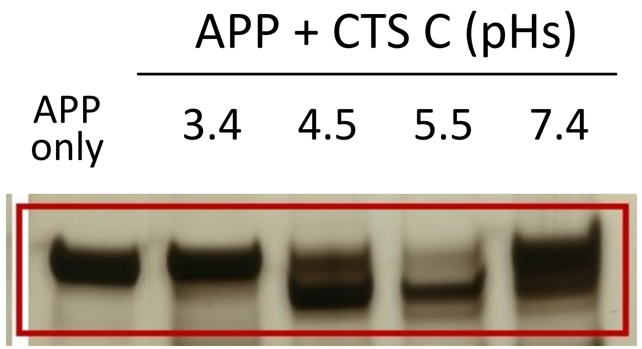

APP: 77 kDa
CTS C: 51 kDa
Glucagon: 13 kDa

Enzymes (1 uM) plus substrate (1ug) incubated at 37C for 1 hour.
Samples run on 4-12% Bis-Tris Gels with MES Buffer and Silver Stained.

Cathepsin D cleaves APP

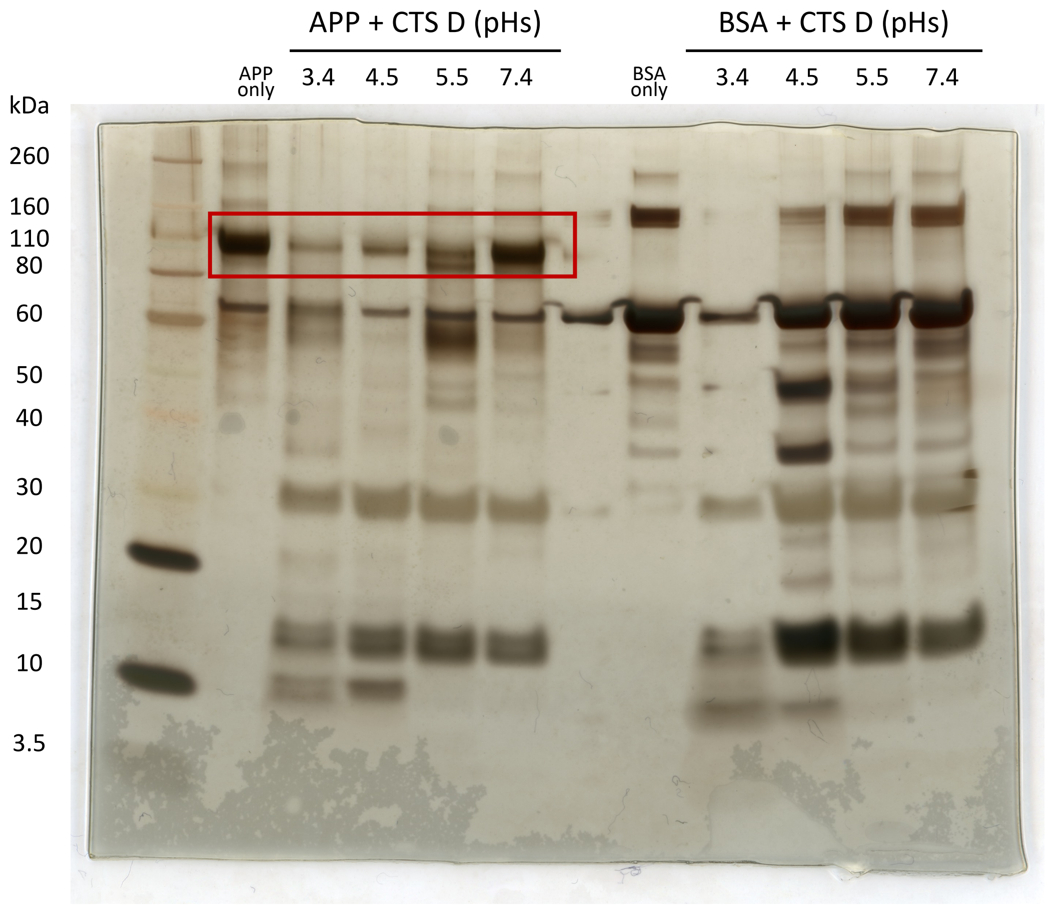

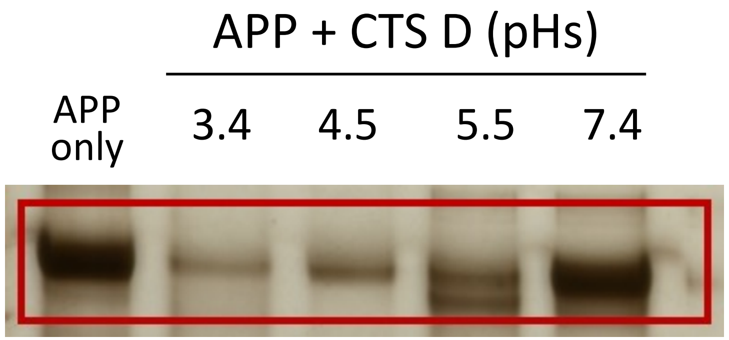

APP: 77 kDa
CTS D: 42 kDa
BSA: 68 kDa

Enzymes (1 uM) plus substrate (1ug) incubated at 37C for 1 hour.
Samples run on 4-12% Bis-Tris Gels with MES Buffer and Silver Stained.

Cathepsin E cleaves APP

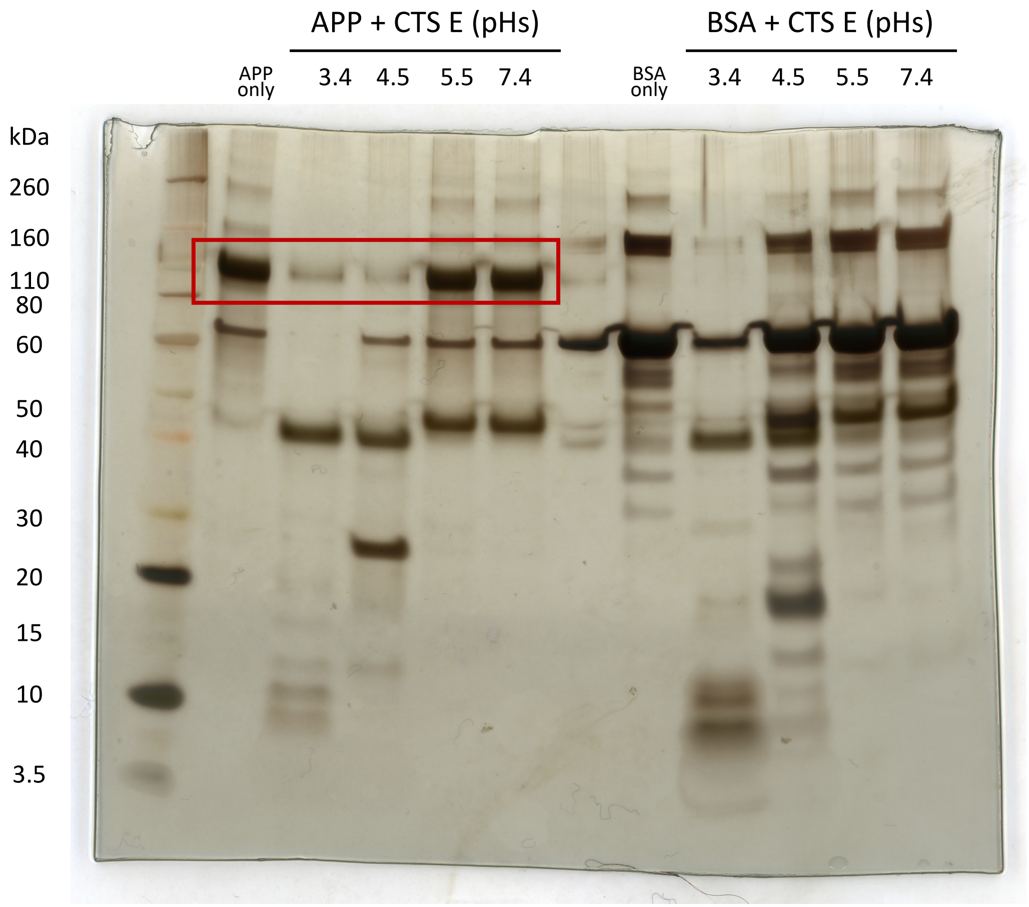

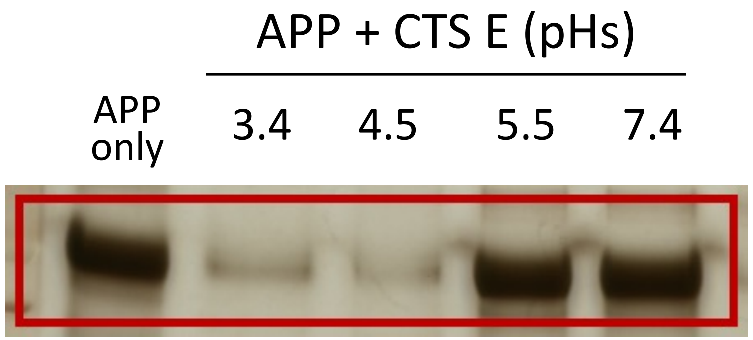

APP: 77 kDa
CTS E: 42 kDa
BSA: 68 kDa

Enzymes (1 uM) plus substrate (1ug) incubated at 37C for 1 hour.
Samples run on 4-12% Bis-Tris Gels with MES Buffer and Silver Stained.

Cathepsin F does not cleave APP

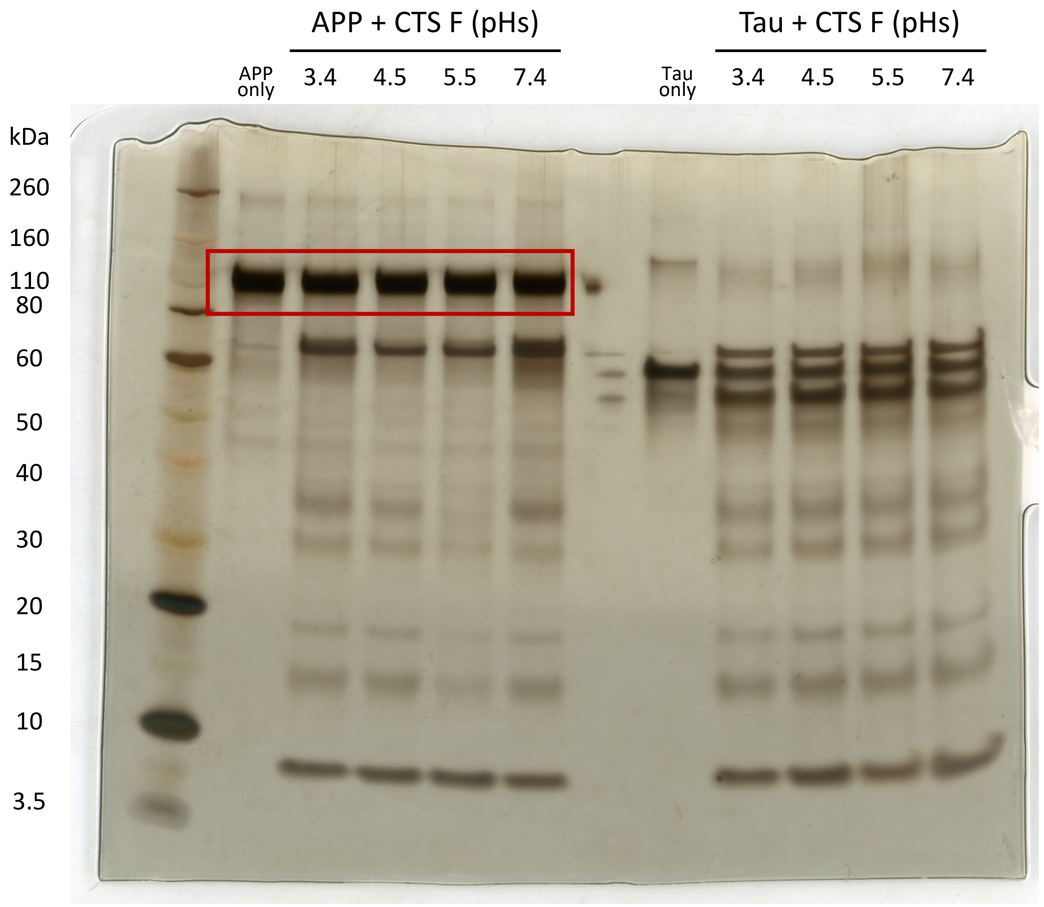

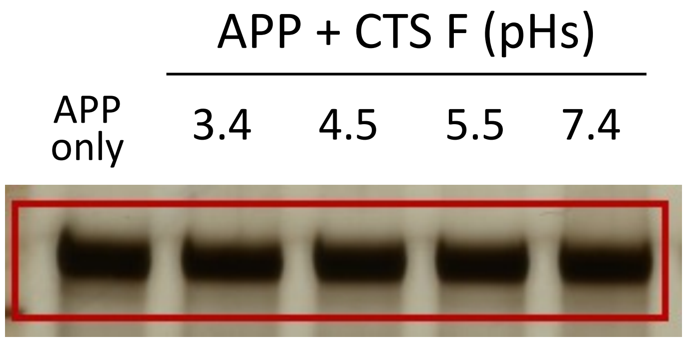

APP: 77 kDa

CTS F: 39 kDa

Tau:

Enzymes (1 uM) plus substrate (1ug) incubated at 37C for 1 hour.
Samples run on 4-12% Bis-Tris Gels with MES Buffer and Silver Stained.

Cathepsin G cleaves APP

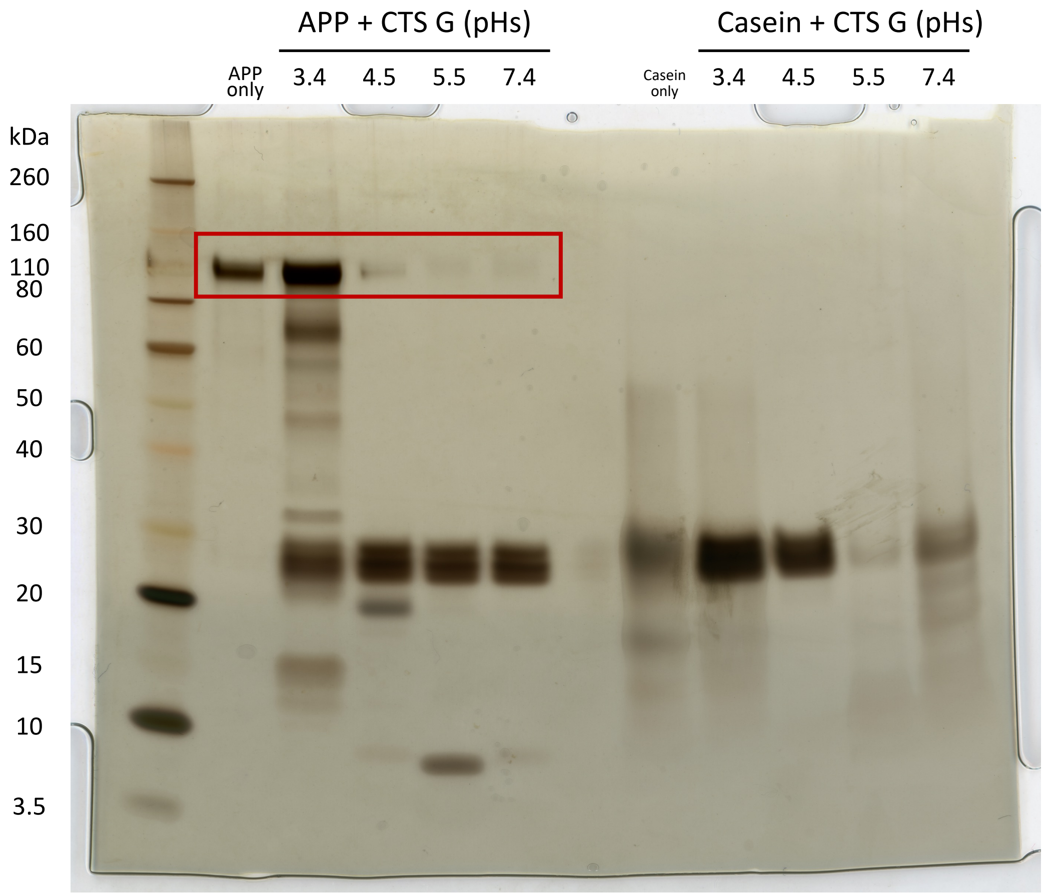

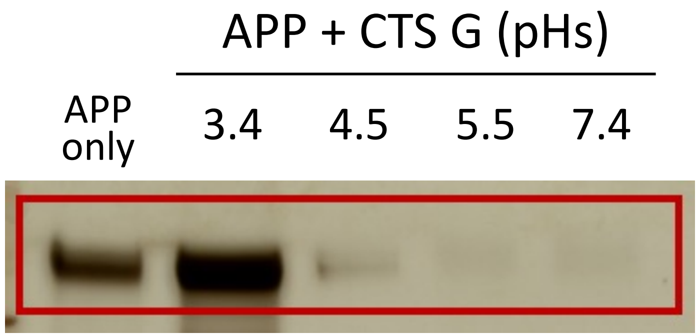

APP: 77 kDa
CTS G: 23.5 kDa
Casein: 24 kDa

Enzymes (1 uM) plus substrate (1ug) incubated at 37C for 1 hour.
Samples run on 4-12% Bis-Tris Gels with MES Buffer and Silver Stained.

Cathepsin H cleaves APP

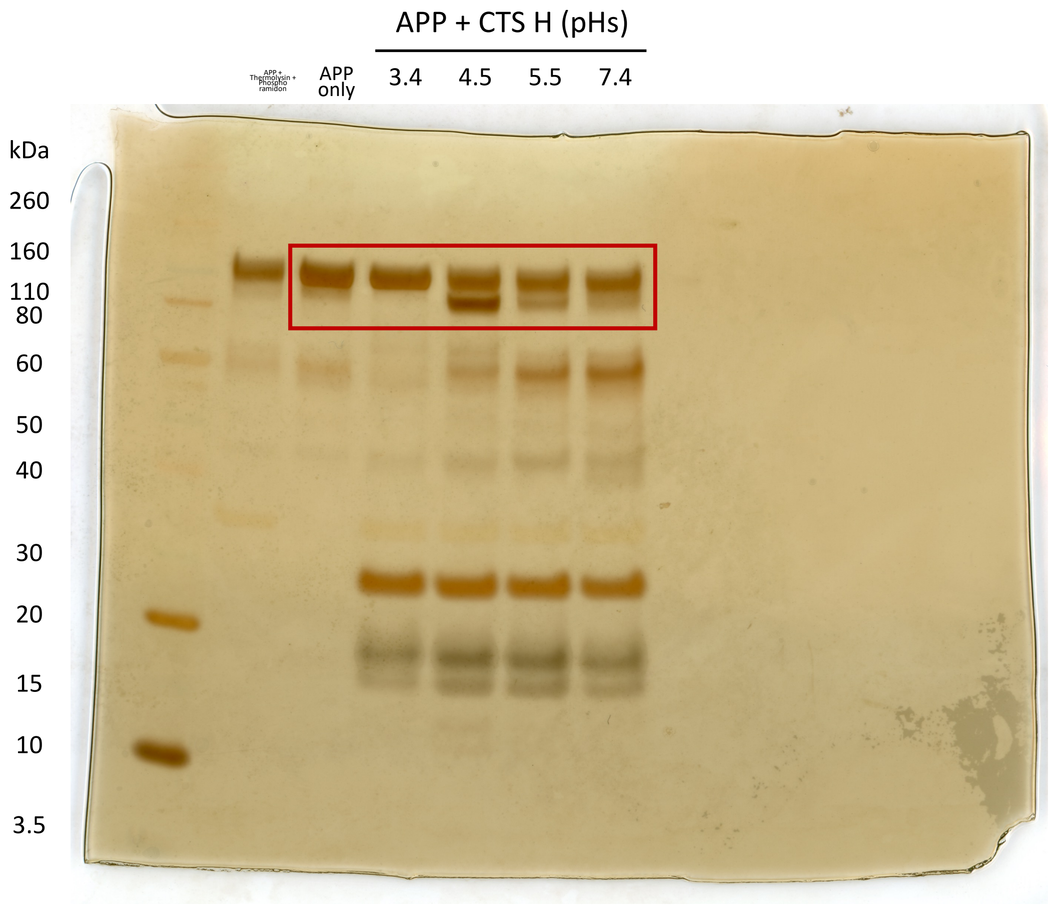

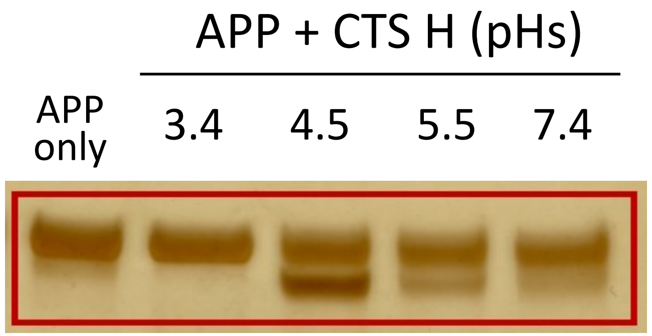

APP: 77 kDa
CTS H: 37 kDa

Enzymes (1 uM) plus substrate (1ug) incubated at 37C for 1 hour.
Samples run on 4-12% Bis-Tris Gels with MES Buffer and Silver Stained.

Cathepsin K cleaves APP

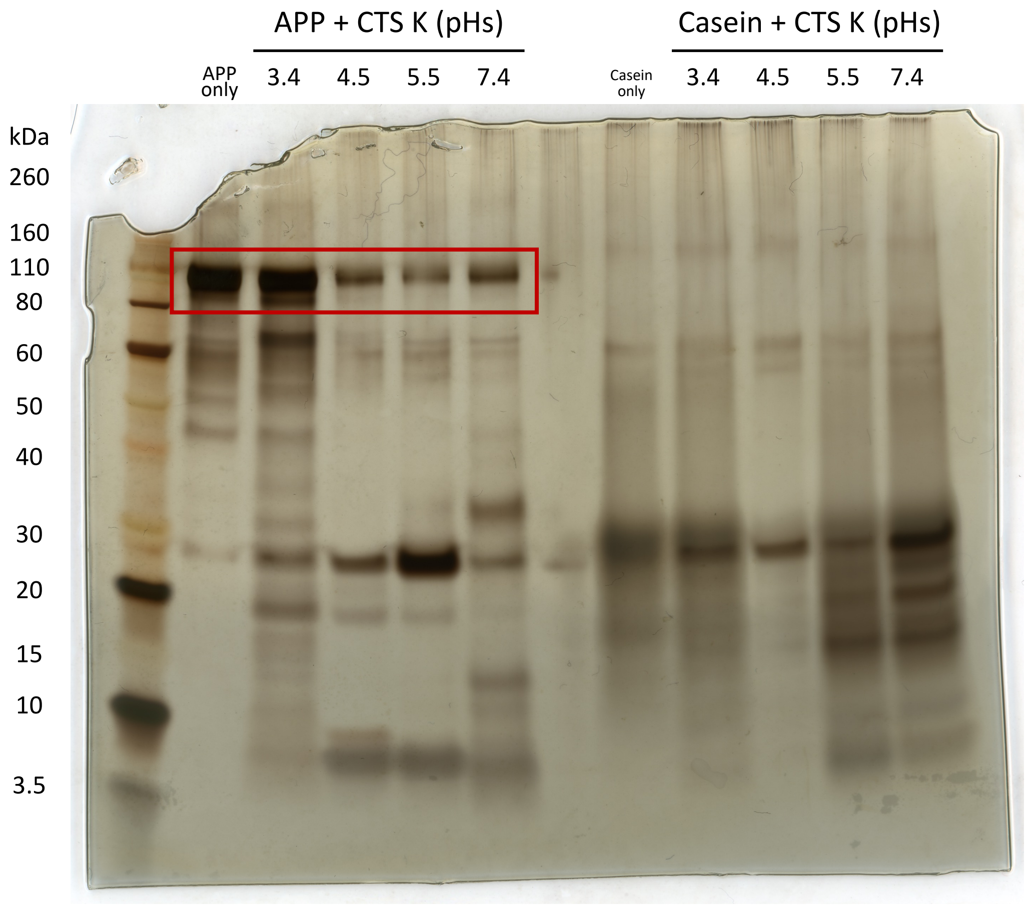

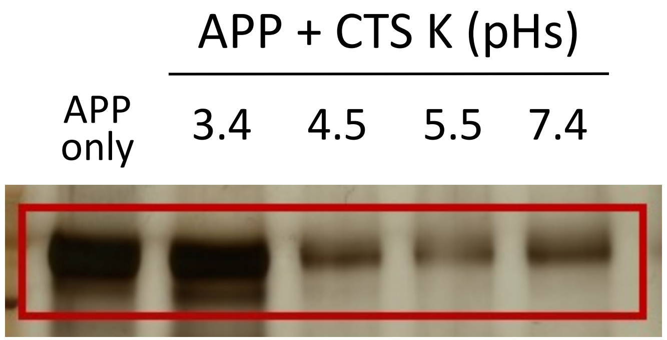

APP: 77 kDa
CTS K: 27 kDa
Casein: 24 kDa

Enzymes (1 uM) plus substrate (1ug) incubated at 37C for 1 hour.
Samples run on 4-12% Bis-Tris Gels with MES Buffer and Silver Stained.

Cathepsin L cleaves APP

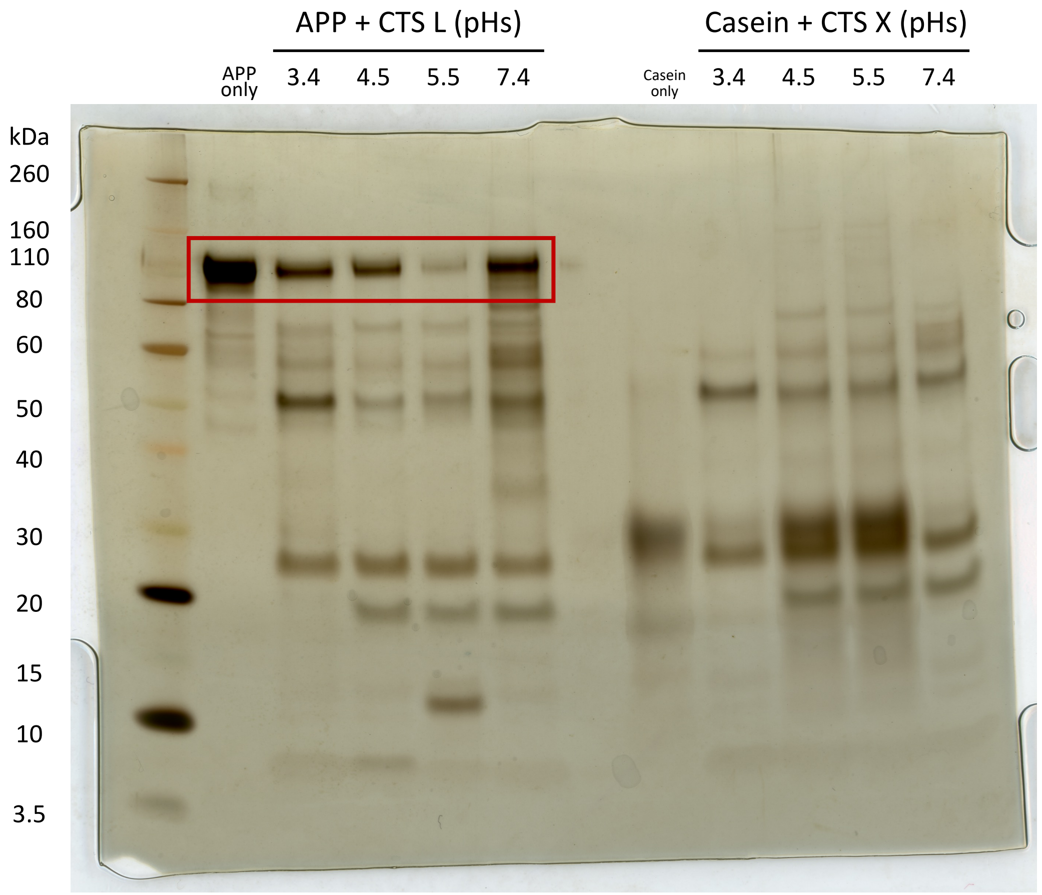

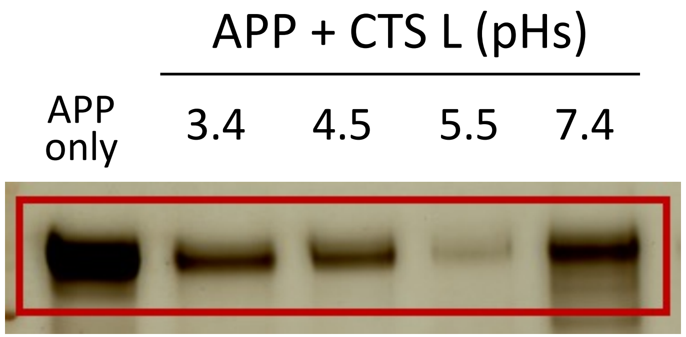

APP: 77 kDa
CTS L: 29 kDa
Casein: 24 kDa

Enzymes (1 uM) plus substrate (1ug) incubated at 37C for 1 hour.
Samples run on 4-12% Bis-Tris Gels with MES Buffer and Silver Stained.

Cathepsin O does not cleave APP

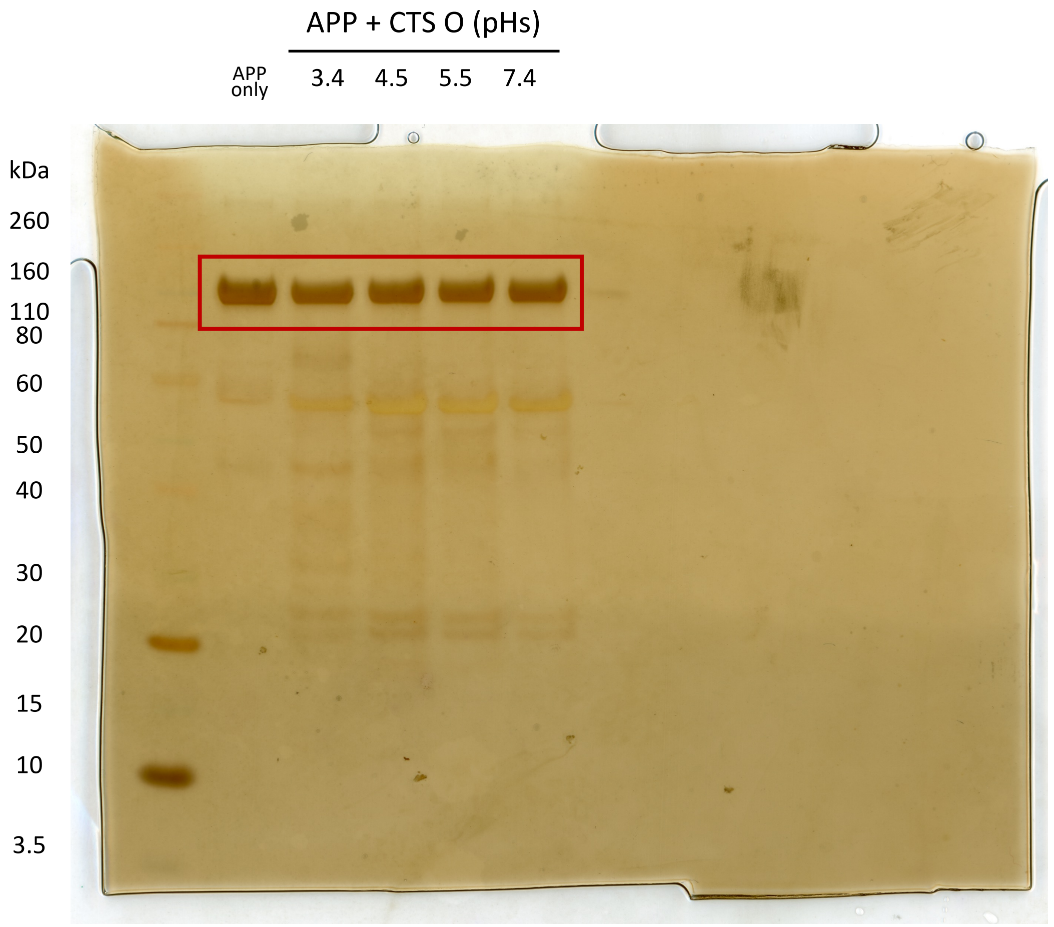

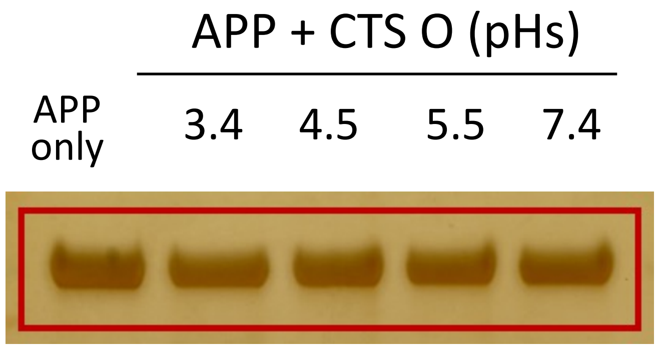

APP: 77 kDa
CTS O: 33 kDa

Enzymes (1 uM) plus substrate (1ug) incubated at 37C for 1 hour.
Samples run on 4-12% Bis-Tris Gels with MES Buffer and Silver Stained.

Cathepsin S cleaves APP

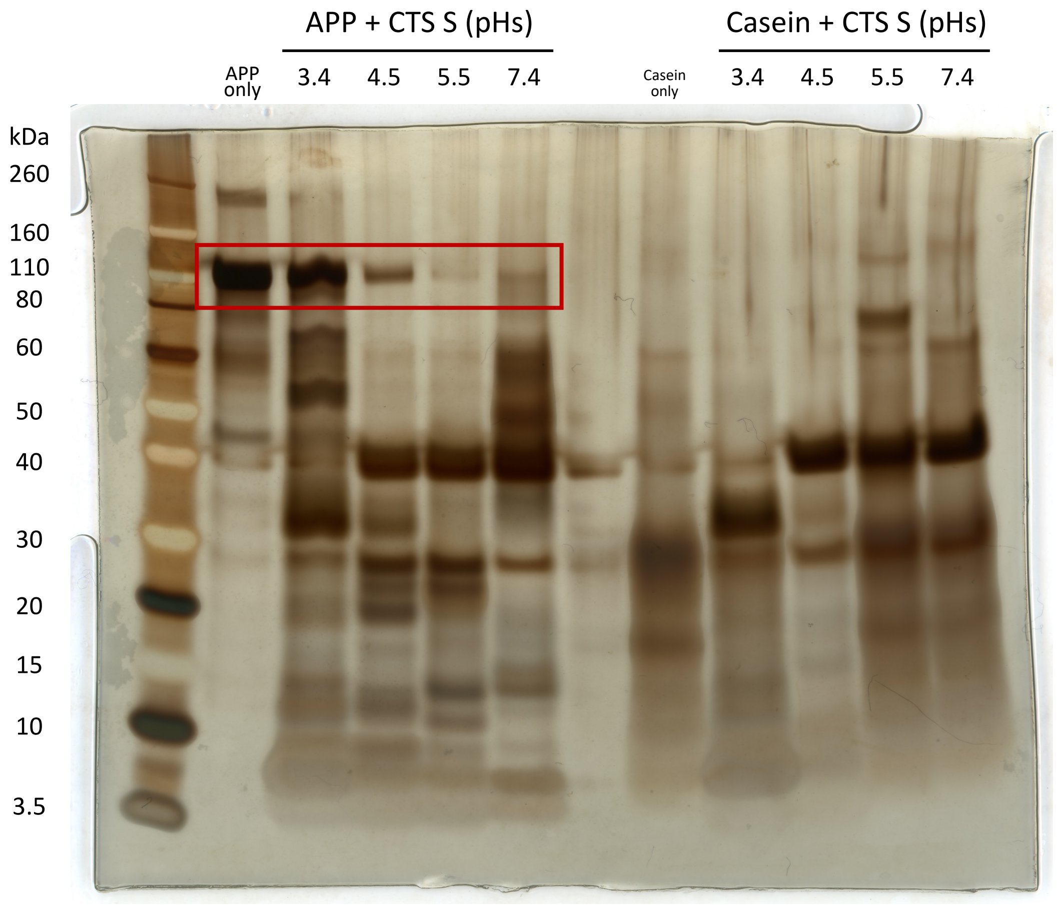

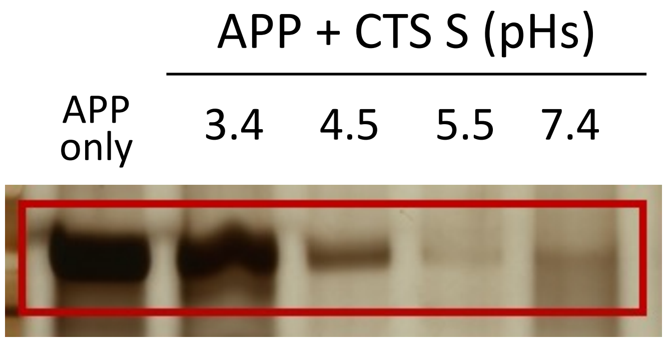

APP: 77 kDa
CTS S: 39 kDa
Casein: 24 kDa

Enzymes (1 uM) plus substrate (1ug) incubated at 37C for 1 hour.
Samples run on 4-12% Bis-Tris Gels with MES Buffer and Silver Stained.

Cathepsin V cleaves APP

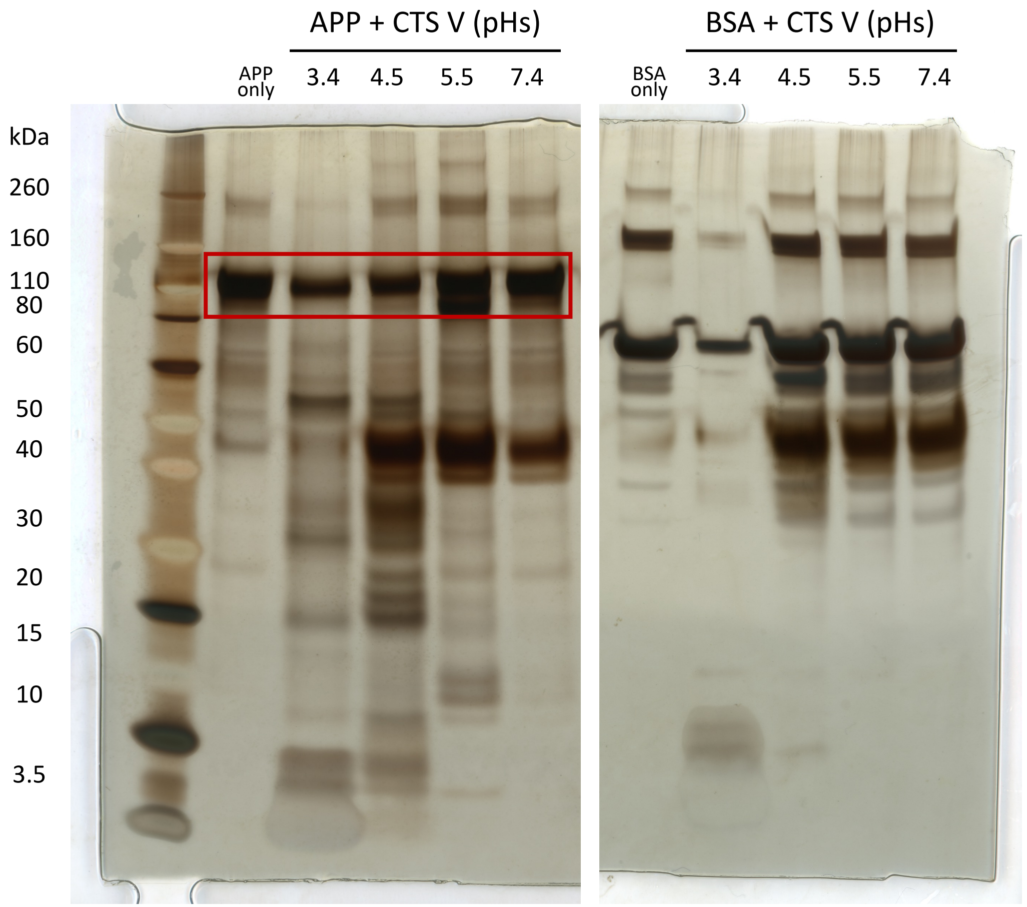

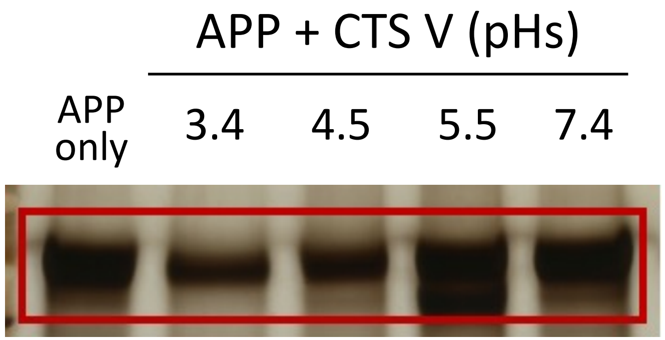

APP: 77 kDa
CTS V: 37 kDa
BSA: 68 kDa

Enzymes (1 uM) plus substrate (1ug) incubated at 37C for 1 hour.
Samples run on 4-12% Bis-Tris Gels with MES Buffer and Silver Stained.

Cathepsin X does not cleave APP

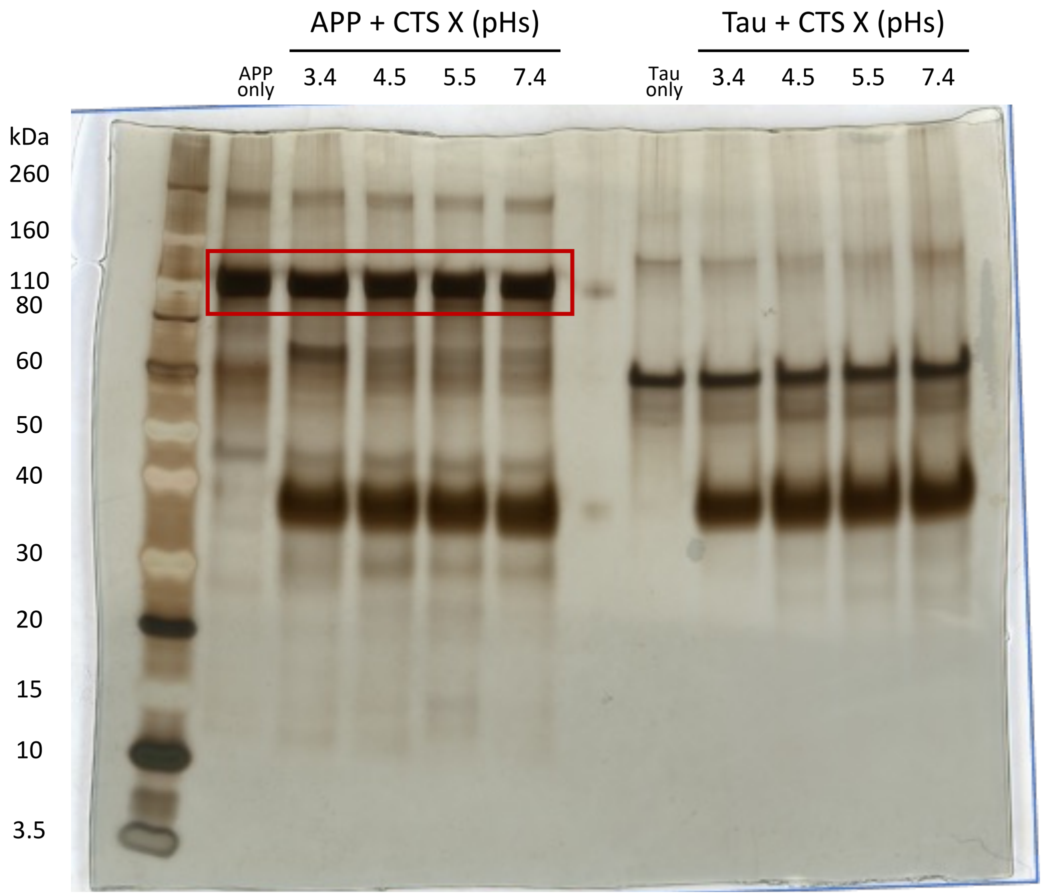

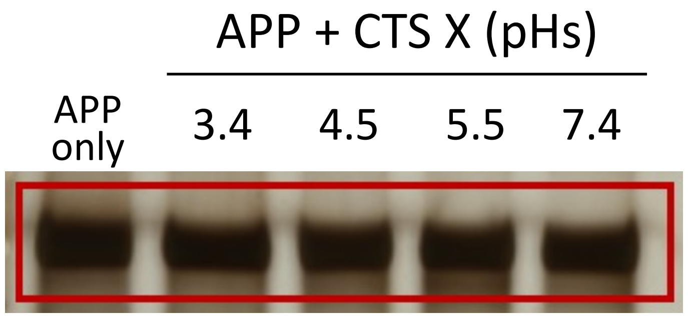

APP: 77 kDa
CTS X: 33 kDa
Tau:

Enzymes (1 uM) plus substrate (1ug) incubated at 37C for 1 hour.
Samples run on 4-12% Bis-Tris Gels with MES Buffer and Silver Stained.

Legumain/AEP cleaves APP

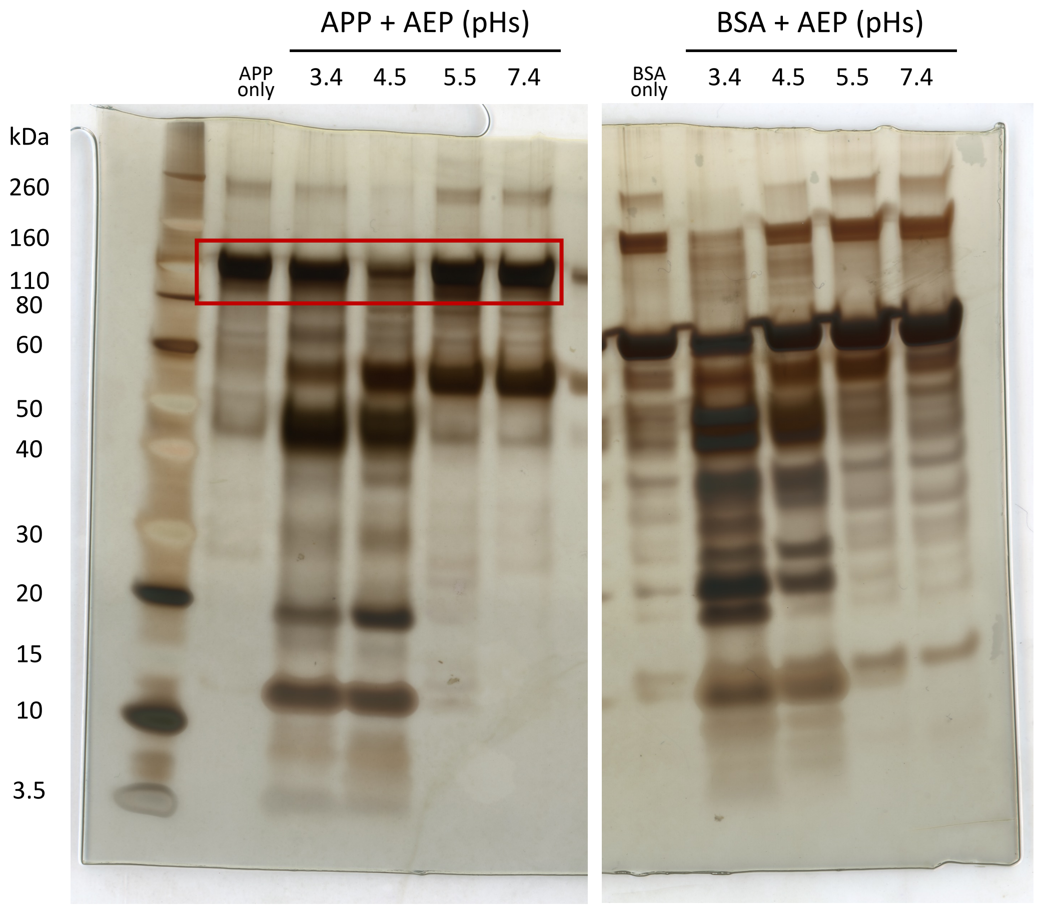

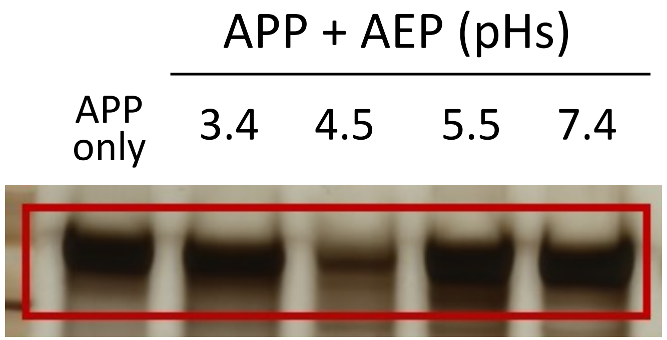

APP: 77 kDa
AEP: 49 kDa
BSA: 68 kDa

Enzymes (1 uM) plus substrate (1ug) incubated at 37C for 1 hour.
Samples run on 4-12% Bis-Tris Gels with MES Buffer and Silver Stained.

Figure S5. Full silver-stained gels demonstrating cathepsin cleavage of sAPP at varying pH values.
Full silver-stained gels from Figure 1E.

Figure S6.

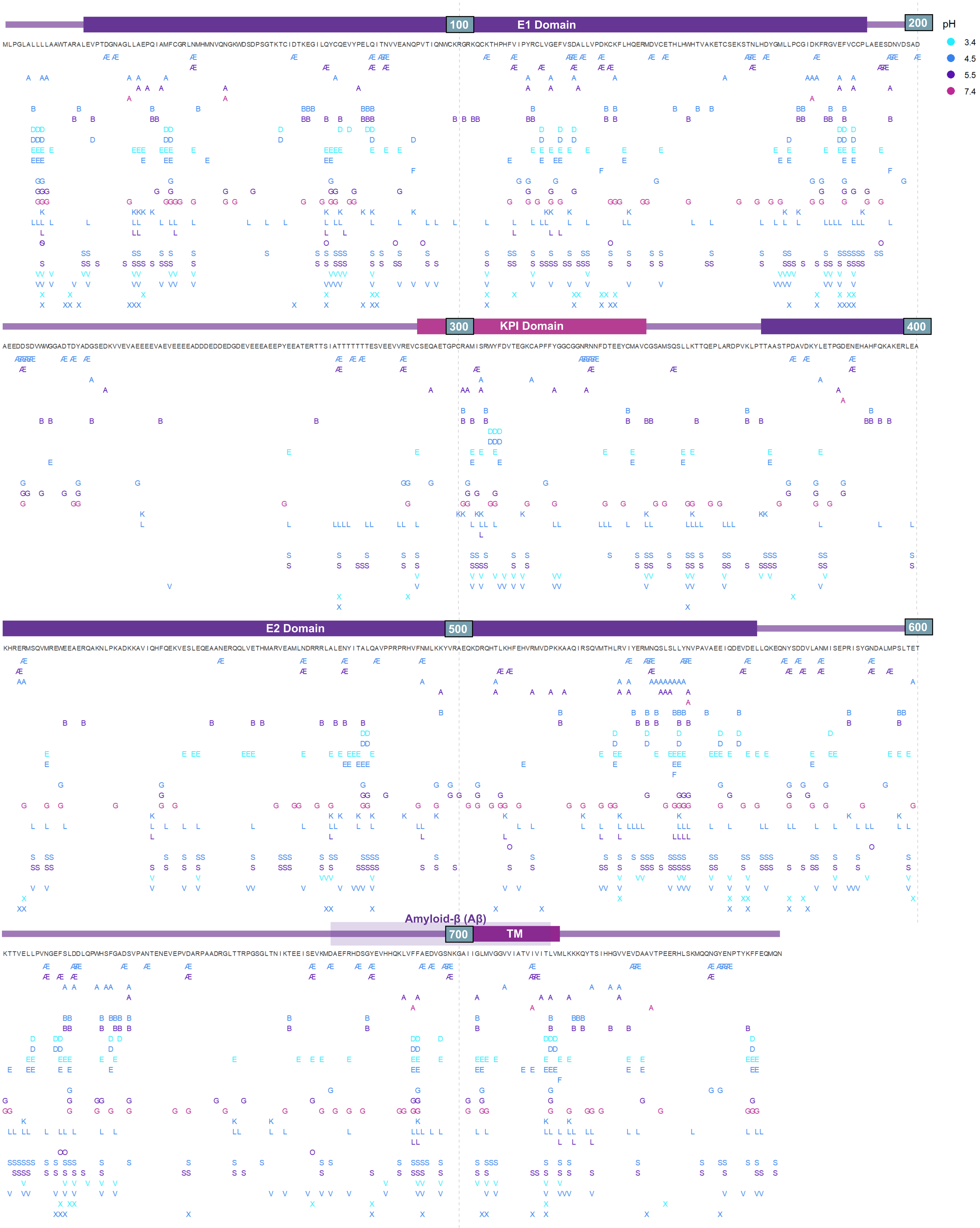

Figure S6. MSP-MS cleavage map of APP of 13 lysosomal proteases at varying pH levels.
A full diagram of cleavage sites identified using MSP-MS including cleavages observed at each pH tested. Letters indicate the P1 site identified for each protease (ie. “G” = CTSG). Æ = legumain/Asparagine Endopeptidase.

Figure S7

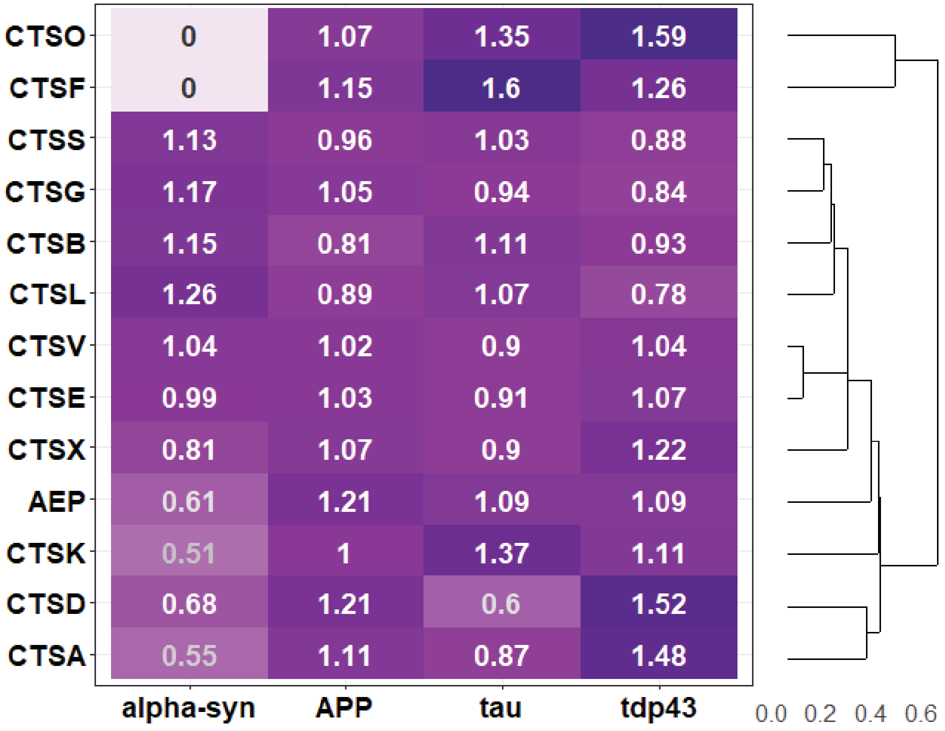

Figure S7. Heirarchical clustering analysis comparing the relative contributions of each enzyme in cleaving neurodegeneration-associated proteins.
A heatmap and dendrogram displaying the relative contributions of each enzyme in cleaving α-synuclein, APP, tau, and TDP43. Higher numbers and darker colors indicate a greater degree of cleavage by a particular enzyme. A dendrogram to the right of the heatmap indicates similarities between enzymes in their relative contributions in cleaving all four proteins.

Figure S8

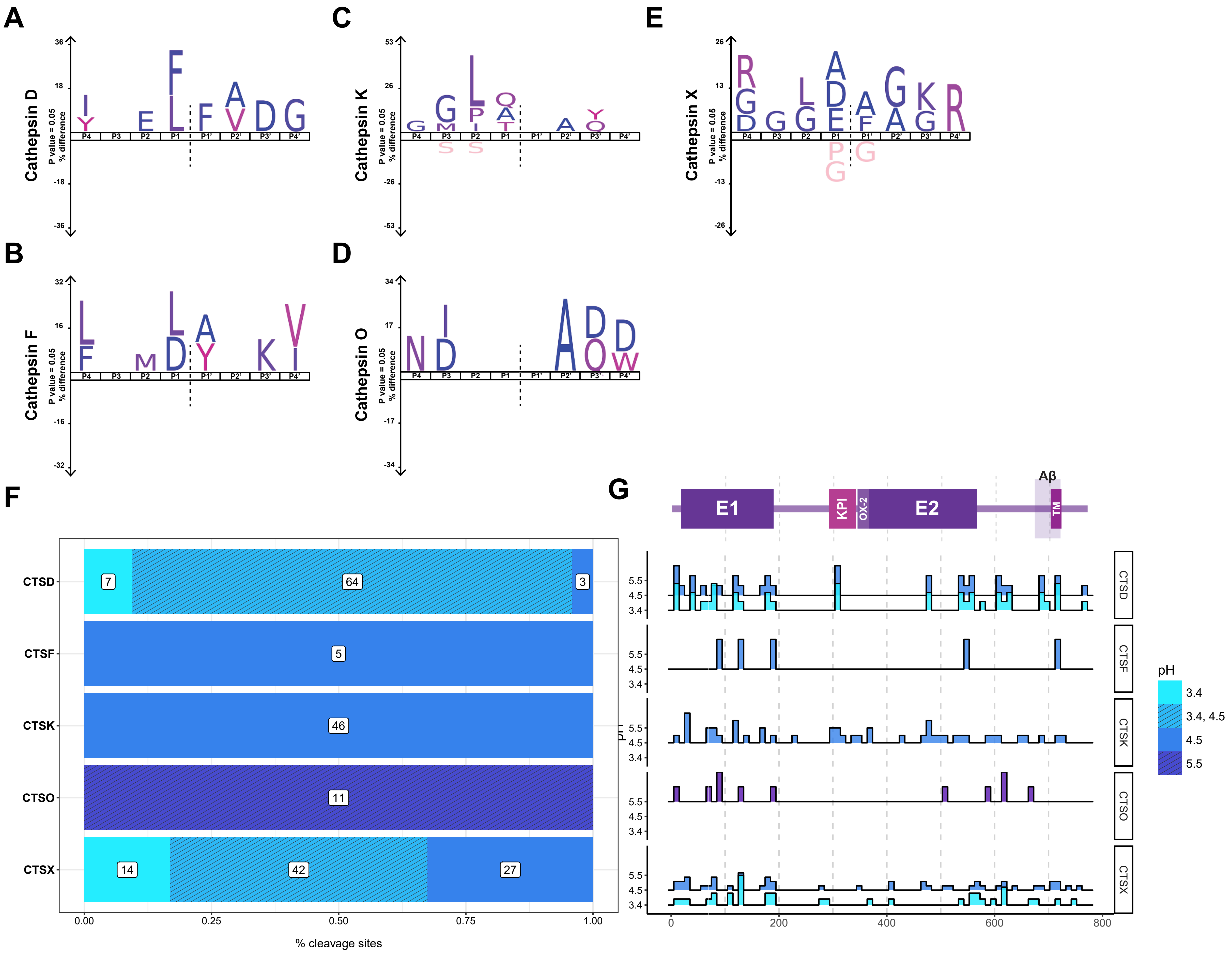

Figure S8. Supplemental IceLogos and cleavage profiles from Figure 3. **(A-D)** IceLogos generated for the remaining enzymes from Figure 3 based on MSP-MS data. **(F)** Stacked barplots representing the number of cleavage sites tallied at each pH. **(G)** Graphical overview of the APP protein and a map of cleavage patterns across APP at varying pH levels. Dotted lines occur every 100 amino acids. E1, E2 = E1 and E2 domains, respectively. KPI = Kunitz Protease Inhibitor domain. TM = transmembrane. Histograms were generated at each experimental pH value and protease combination tested using MSP-MS. Bar heights indicate the relative number of cleavage sites for each condition (ie. the area under each plot = 100%).

Figure S9

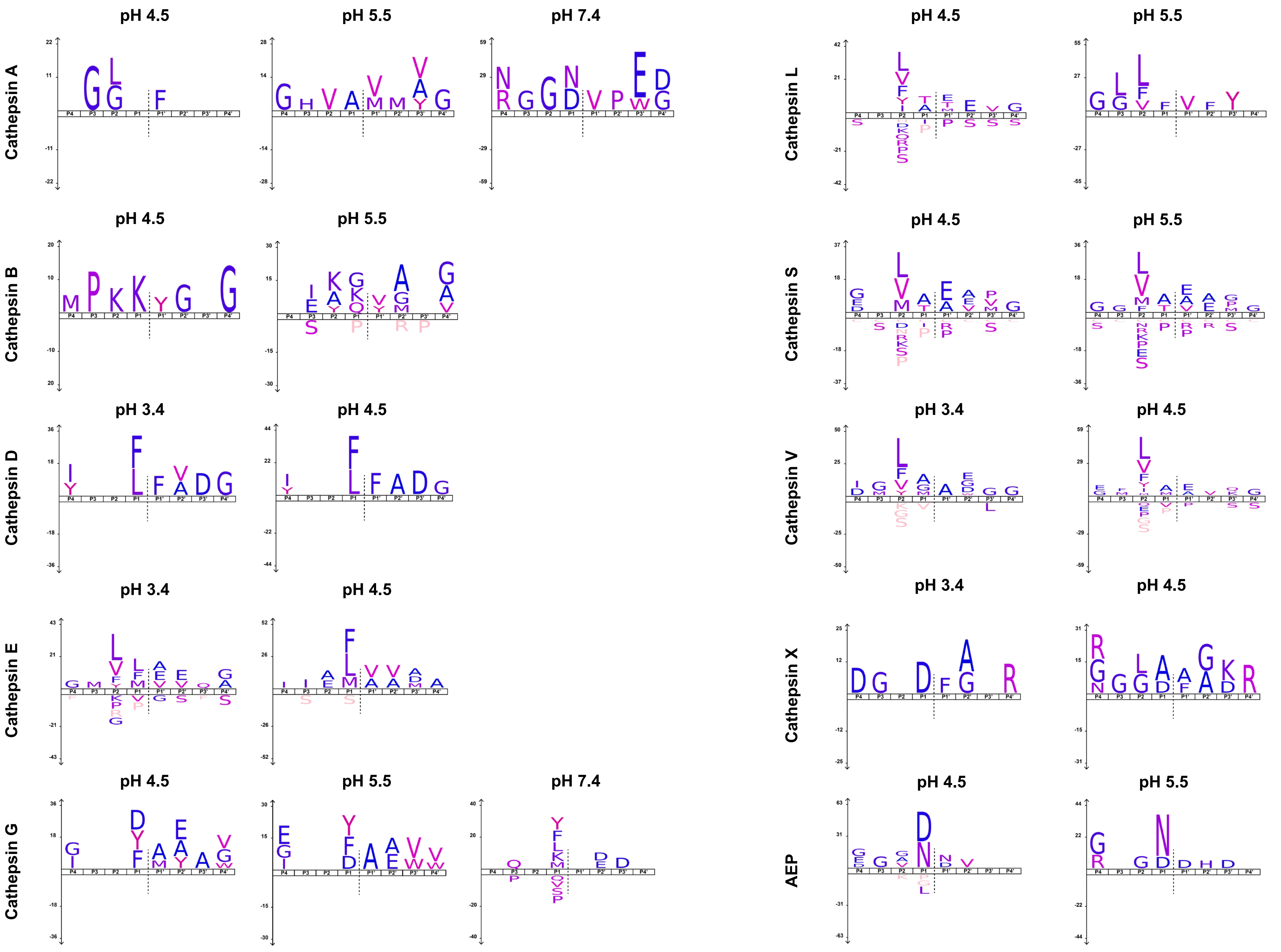

Figure S9. IceLogos of APP MSP-MS data by pH.
IceLogos were generated for each enzyme at every pH tested using MSP-MS (APP data only).

Figure S10

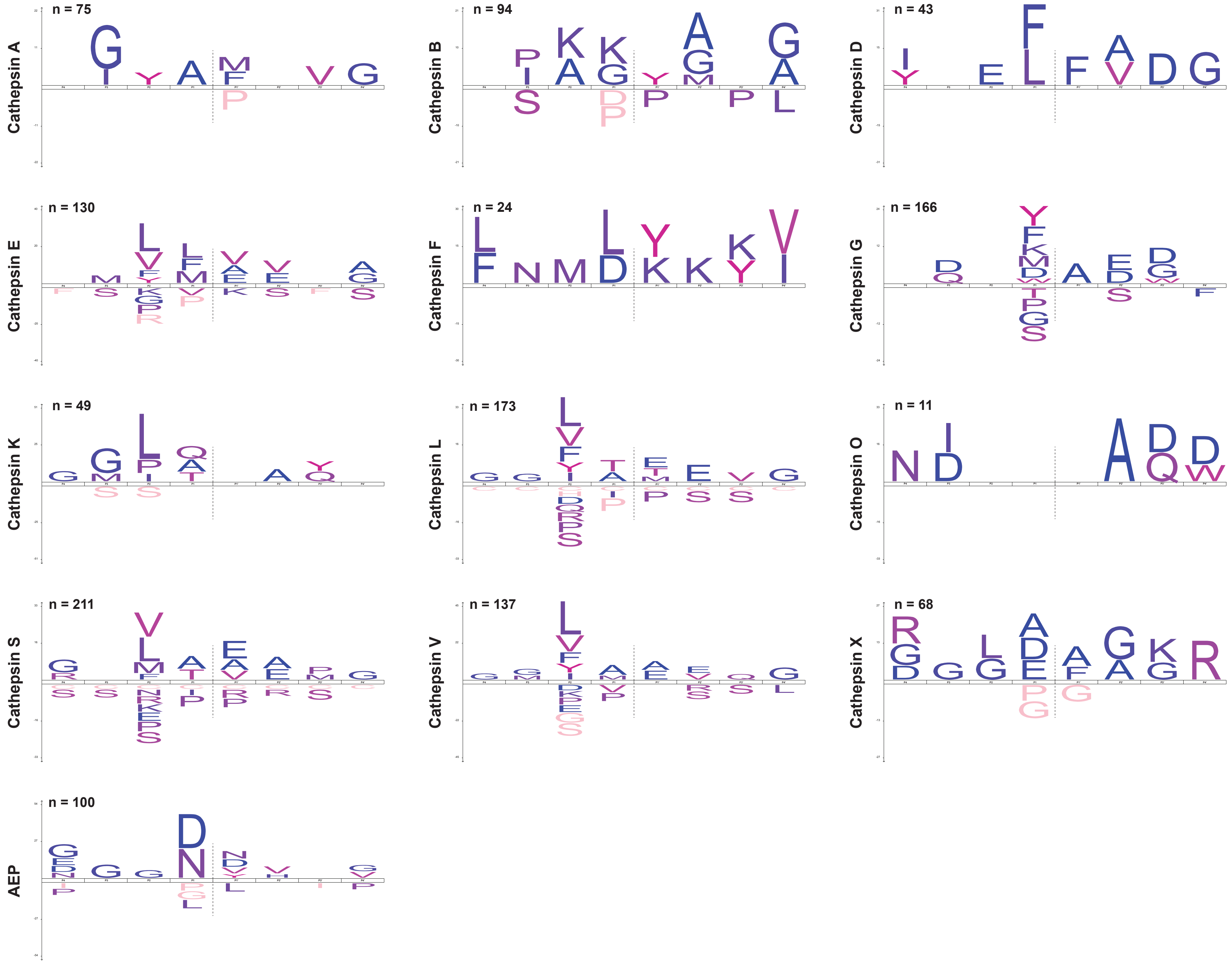

Figure S10. Combined IceLogos of current and previously published MSP-MS datasets. IceLogos were created from a combined MSP-MS dataset including proteolytic cleavage profiles of APP as well as tau, α-synuclein, and TDP43.

Figure S11

A

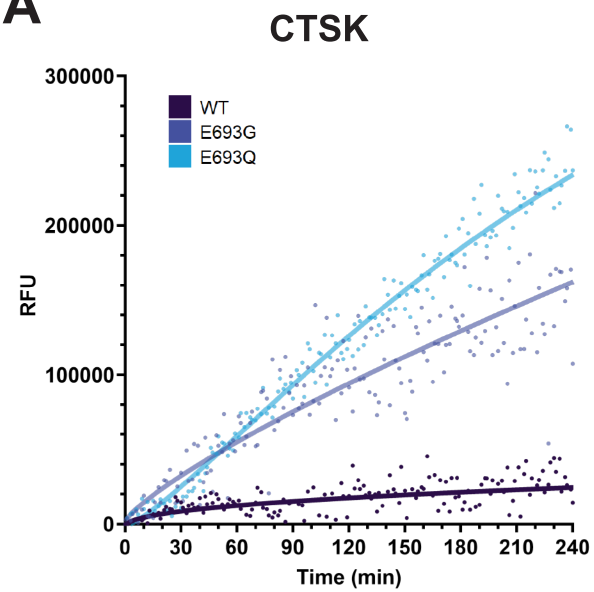

B

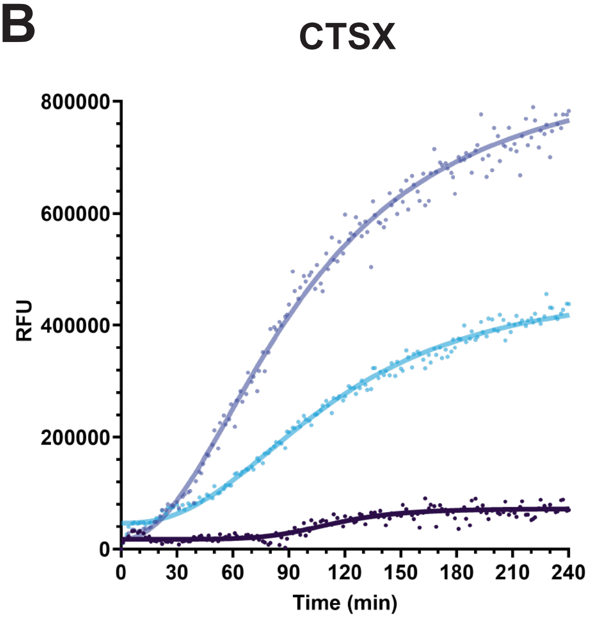

C

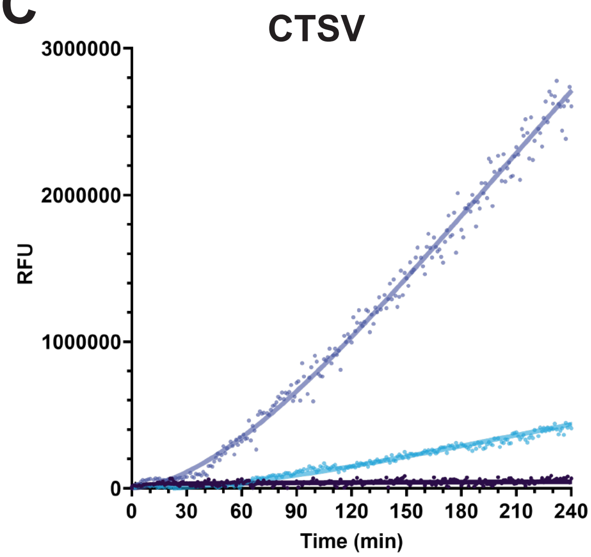

D

	693 WT	E693G	E693Q
CTSB		>99	>99
CTSD	1.00	8.28	6.57
CTSE	1.00	14.04	4.20
CTSG		>99	>99
CTSK		>99	>99
CTSL		>99	>99
CTSS		>99	>99
CTSV		>99	>99
CTSX	1.00	8.40	3.73

Figure S11. Supplemental fluorogenic peptide cleavage assays from Figure 4. **(A–C)** Activity profiles for supplemental enzymes in cleaving wildtype and E693 variant peptides. **(D)** Vmax values for each enzyme in cleaving E693, E693G, and E693Q fluorogenic peptides. Grey boxes denote instances in which no cleavage occured.

Figure S12

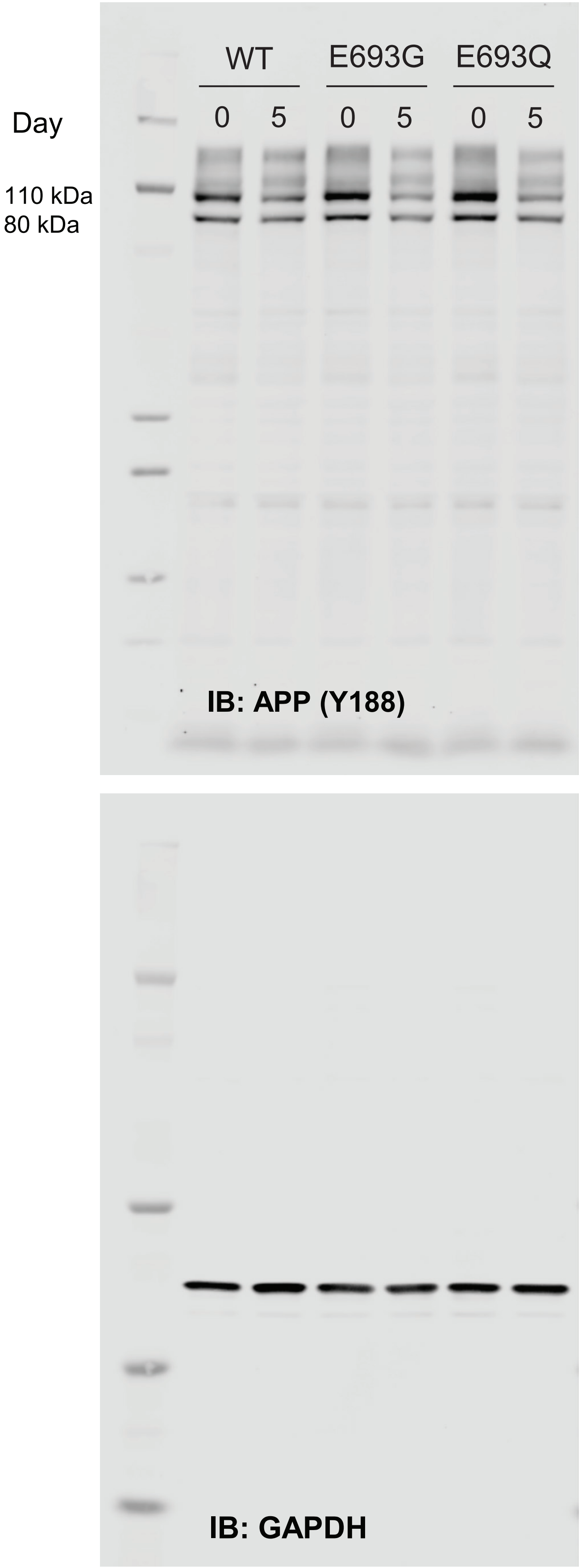

Figure S12. Full blots from Figure 4.
Full Western blot images of wildtype and E693 variant SH-SY5Y lysates (Figure 4I).

Figure S13

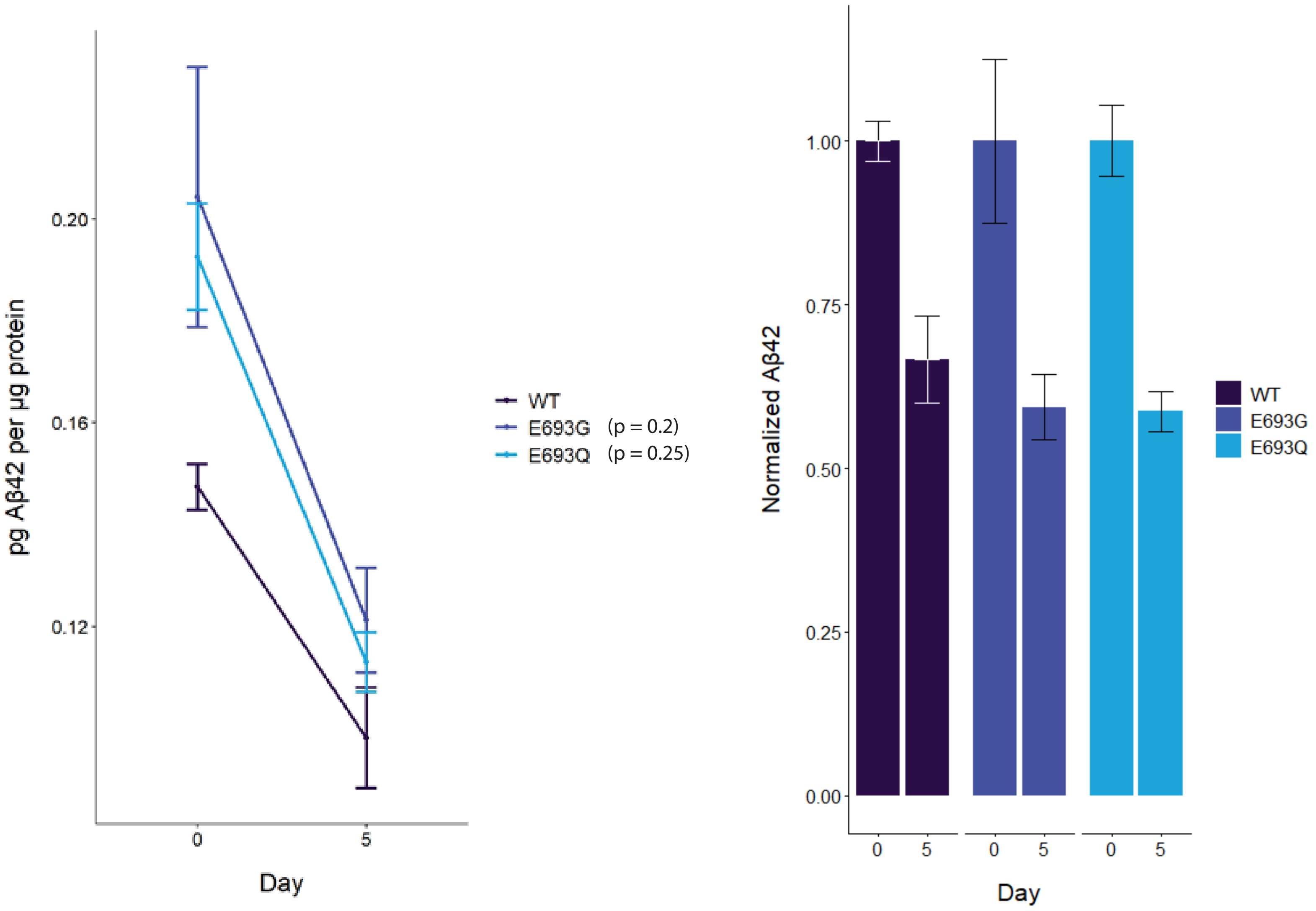

Figure S13. Aβ-42 decreases more rapidly in E693G/Q differentiated SH-SY5Y neurons similar to APP, although this trend is not significant
Cell lysates from Figure 4 were compared using an ELISA against Aβ-42. Error bars represent +/- SEM.

Figure S14

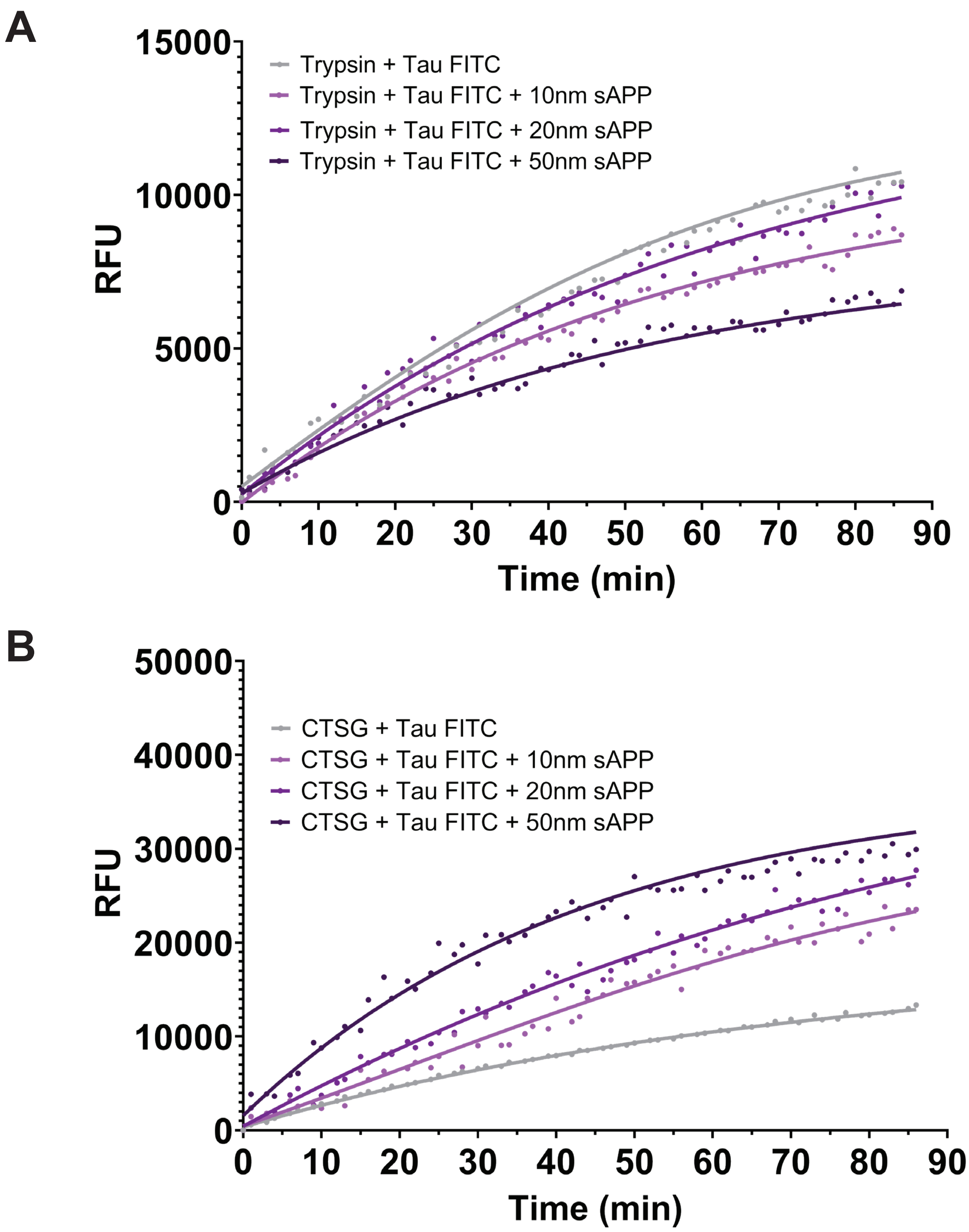

Figure S14. Increasing concentrations of sAPP enhance CTSG cleavage of FITC tau and inhibit its cleavage by Trypsin.
(A) Trypsin cleavage of FITC labelled tau was observed in the presence of increasing concentrations of sAPP**(B)** CTSG cleavage of FITC labelled tau was observed in the presence of increasing concentrations of sAPP.

Figure S15

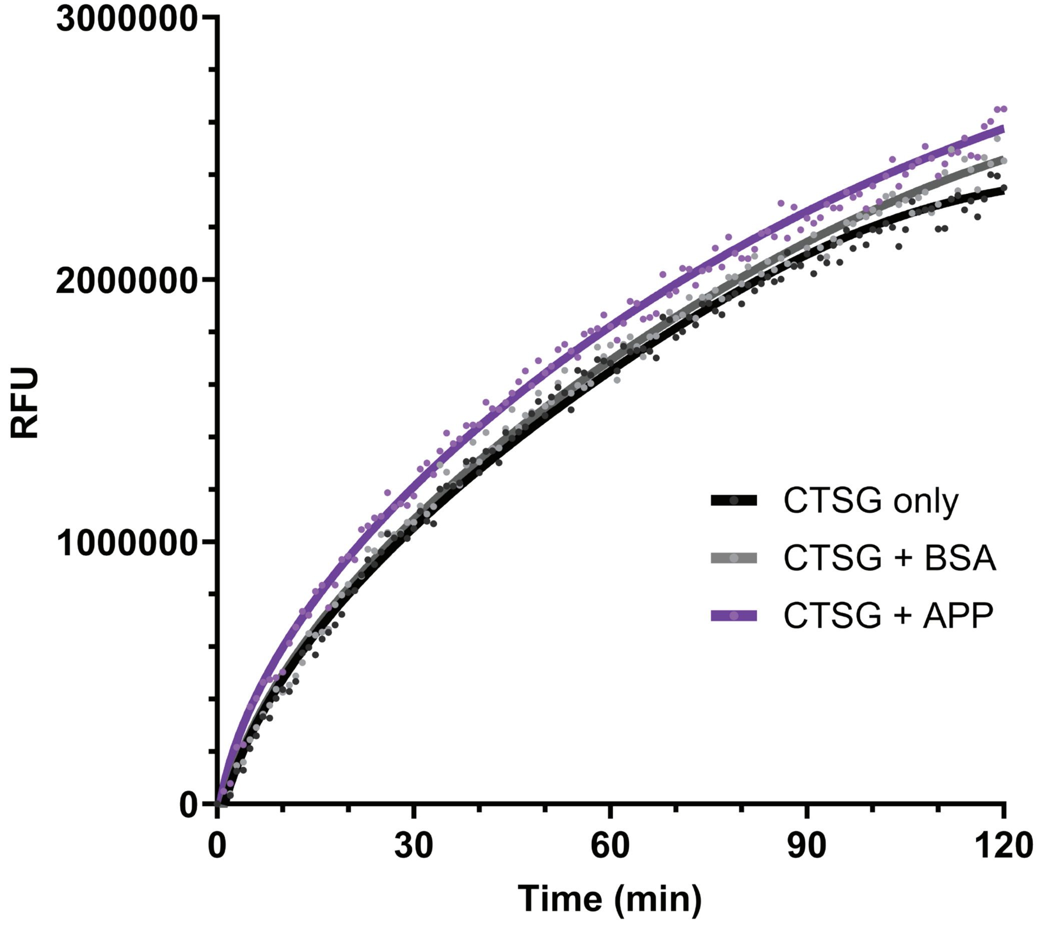

Figure S15. APP does not alter cleavage of a universal substrate by cathepsin G. Activity assay plotting cleavage of a fluorogenic universal protein substrate by cathepsin G in the presence of either BSA or APP. Cleavage activity was determined by quantifying fluorescence over time.

Figure S16

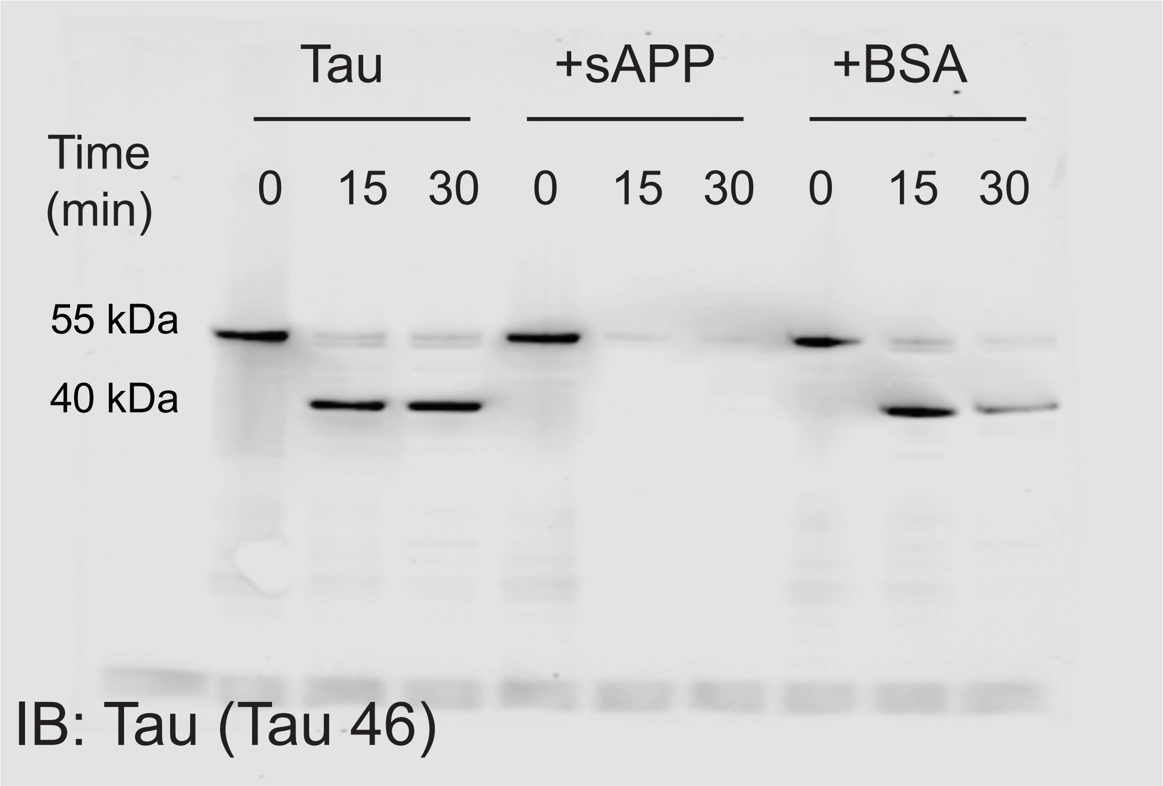

Figure S16. Full blot from Figure 5.
Full Western blot from an *in vitro* cleavage assay of tau by cathepsin G in the presence of either sAPP or BSA (Figure 5D).

Figure S17

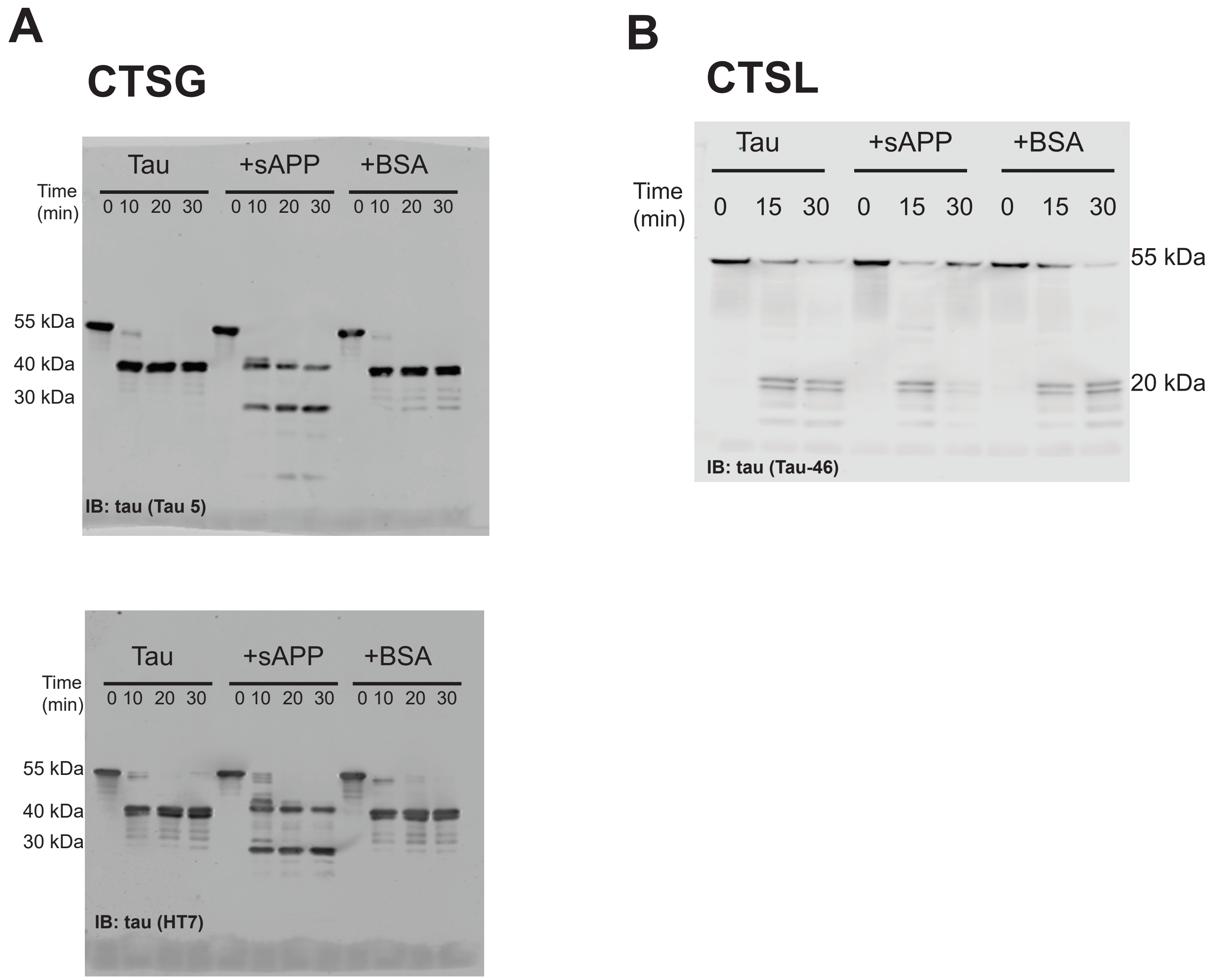

Figure S17. sAPP augments CTSG, but not CTSL, cleavage of tau
Proteolytic cleavage experiments were performed as in Figure 5. **A)** A CTSG/tau cleavage assay Western Blot (with samples collected at 0, 10, 20, and 30 minutes) was stripped and reprobed with Tau 5 and HT7 antibodies, which bind to the proline-rich domain of Tau. **B)** A similar tau cleavage assay was also performed using CTSL.
